# Escalate and De-Escalate Therapies for Intracranial Pressure Control in Traumatic Brain Injury

**DOI:** 10.3389/fneur.2020.564751

**Published:** 2020-11-24

**Authors:** Denise Battaglini, Pasquale Anania, Patricia R. M. Rocco, Iole Brunetti, Alessandro Prior, Gianluigi Zona, Paolo Pelosi, Pietro Fiaschi

**Affiliations:** ^1^Department of Anesthesia and Intensive Care, Ospedale Policlinico San Martino, Istituto di Ricovero e Cura a Carattere Scientifico (IRCCS) for Oncology and Neuroscience, Genoa, Italy; ^2^Department of Neurosurgery, Ospedale Policlinico San Martino, Istituto di Ricovero e Cura a Carattere Scientifico (IRCCS) for Oncology and Neuroscience, Genoa, Italy; ^3^Laboratory of Pulmonary Investigation, Carlos Chagas Filho Biophysics Institute, Federal University of Rio de Janeiro, Rio de Janeiro, Brazil; ^4^Rio de Janeiro Network on Neuroinflammation, Carlos Chagas Filho Foundation for Supporting Research in the State of Rio de Janeiro (FAPERJ), Rio de Janeiro, Brazil; ^5^Rio de Janeiro Innovation Network in Nanosystems for Health—Nano SAÚDE/Carlos Chagas Filho Foundation for Supporting Research in the State of Rio de Janeiro (FAPERJ), Rio de Janeiro, Brazil; ^6^Department of Neuroscience, Rehabilitation, Ophthalmology, Genetics and Maternal and Child Health (DINOGMI), University of Genoa, Genoa, Italy; ^7^Department of Surgical Sciences and Integral Diagnostics (DISC), University of Genoa, Genoa, Italy

**Keywords:** trauma, intracranial hypertension (ICH), escalation, traumatic brain injury, staircase algorithm

## Abstract

Severe traumatic brain injury (TBI) is frequently associated with an elevation of intracranial pressure (ICP), followed by cerebral perfusion pressure (CPP) reduction. Invasive monitoring of ICP is recommended to guide a step-by-step “staircase approach” which aims to normalize ICP values and reduce the risks of secondary damage. However, if such monitoring is not available clinical examination and radiological criteria should be used. A major concern is how to taper the therapies employed for ICP control. The aim of this manuscript is to review the criteria for escalating and withdrawing therapies in TBI patients. Each step of the staircase approach carries a risk of adverse effects related to the duration of treatment. Tapering of barbiturates should start once ICP control has been achieved for at least 24 h, although a period of 2–12 days is often required. Administration of hyperosmolar fluids should be avoided if ICP is normal. Sedation should be reduced after at least 24 h of controlled ICP to allow neurological examination. Removal of invasive ICP monitoring is suggested after 72 h of normal ICP. For patients who have undergone surgical decompression, cranioplasty represents the final step, and an earlier cranioplasty (15–90 days after decompression) seems to reduce the rate of infection, seizures, and hydrocephalus.

## Introduction

Traumatic brain injury (TBI) is a major public health problem, affecting ~64–74 million people and causing 5 million deaths every year, although its true impact seems to be underestimated owing to incomplete data from developing countries (1). TBI carries high rates of hospitalization, morbidity, and mortality. Its pathophysiology is characterized by an elevation of intracranial pressure (ICP), followed by a reduction in cerebral perfusion pressure (CPP) with possible secondary brain damage (2, 3). Monitoring of ICP and surveillance of risk factors for secondary brain injury is recommended by international guidelines (2–4), despite a randomized multicenter international trial investigating monitored and non-monitored patients did not reveal substantial differences in term of outcome (5). Besides, 23–89% of patients are managed without ICP monitoring both for limited resources and expertise, although this can occur also in high-resource countries (6, 7).

A step-by-step approach to treatment escalation, known as the “staircase approach” (3), aiming to obtain normal ICP values and adequate CPP as well as to reduce the risks of secondary damage is recommended for ICP management in patients who present an invasive ICP (inv-ICP) monitoring device (3, 4). Otherwise, in case of non-availability of ICP monitoring, the SIBICC Consensus Protocol for escalating treatments should be followed (6). Hence, two different approaches have been described to manage severe TBI patients, depending on the standard of care, resources-limit, and expertise: (1) pursuing the indications of inv-ICP monitoring, or (2) following brain imaging and clinical examination to escalate therapies. Even though inv-ICP monitoring is not easy to manage, it is recommended by most guidelines (3, 4, 6, 8, 9). Concerning inv-ICP placement, the Brain Trauma Foundation (BTF) and the 2019 SIBICC Consensus Conference leave the decision to the clinician, because previous recommendations were not as strong as needed—previous indications included patients with pathological findings on computed tomography (CT) and a Glasgow Coma Score (GCS) < 8, or impossibility to perform the neurological examination, and patients with normal CT-scan with unavailable neurological examination and two or more of the following risk factors: age > 40 years, hypotension, and abnormal flexion/extension in response to pain (4, 10). TBI is frequently complicated by HICP, which is defined as an increase in ICP over 20–22 mmHg (in inv-ICP monitored patients) (3, 4), while in non-invasively monitored patients who are managed according to imaging and clinical criteria, HICP can be suspected when one major or two minor criteria are met. Major criteria include compressed cisterns (CT classification of Marshall diffuse injury III), midline shift of more than 5 mm (CT classification of Marshall diffuse injury IV), and non-evacuated mass; minor criteria include GCS motor score ≤ 4, pupillary asymmetry, altered pupillary reactivity, midline shift 0–5 mm, and/or lesion of 25 or less cm^3^ (CT classification of Marshall diffuse injury II). The risk of not monitoring ICP could be an overtreatment of patients with acceptable ICP and an undertreatment of patients with potentially harmful HICP (6, 11). Refractory HICP is defined as intracranial pressure that exceeds 22–25 mmHg for 30 min, or 30 mmHg for 15 min, or 40 mmHg for 1 min (12), and this is the recommended ICP threshold to pursue more aggressive therapies (3, 4). According to the most recent guidelines for the management of TBI, the treatment of HICP is divided into several steps, until the most aggressive including surgical decompression (4, 9, 10). A main concern in neurointensive care unit practice remains how to manage and de-escalate the employed therapies once ICP and CPP targets have been achieved. In fact, each step of treatment escalation carries potential side effects (e.g., hypotension, infection, pneumonia, brain ischemia, electrolyte, and fluid disturbances), frequently related to the duration of treatment (3). Although the management of intracranial hypertension has been widely explored in literature, little evidence is available for withdrawing these treatments and returning to baseline condition.

Therefore, the aim of our narrative review is to briefly describe current practice for the management of intracranial hypertension and to analyze how and when it is recommended to de-escalate HICP therapies in patients with severe TBI, with or without inv-ICP monitoring.

## ICP Pathophysiology

**Figure 1:**
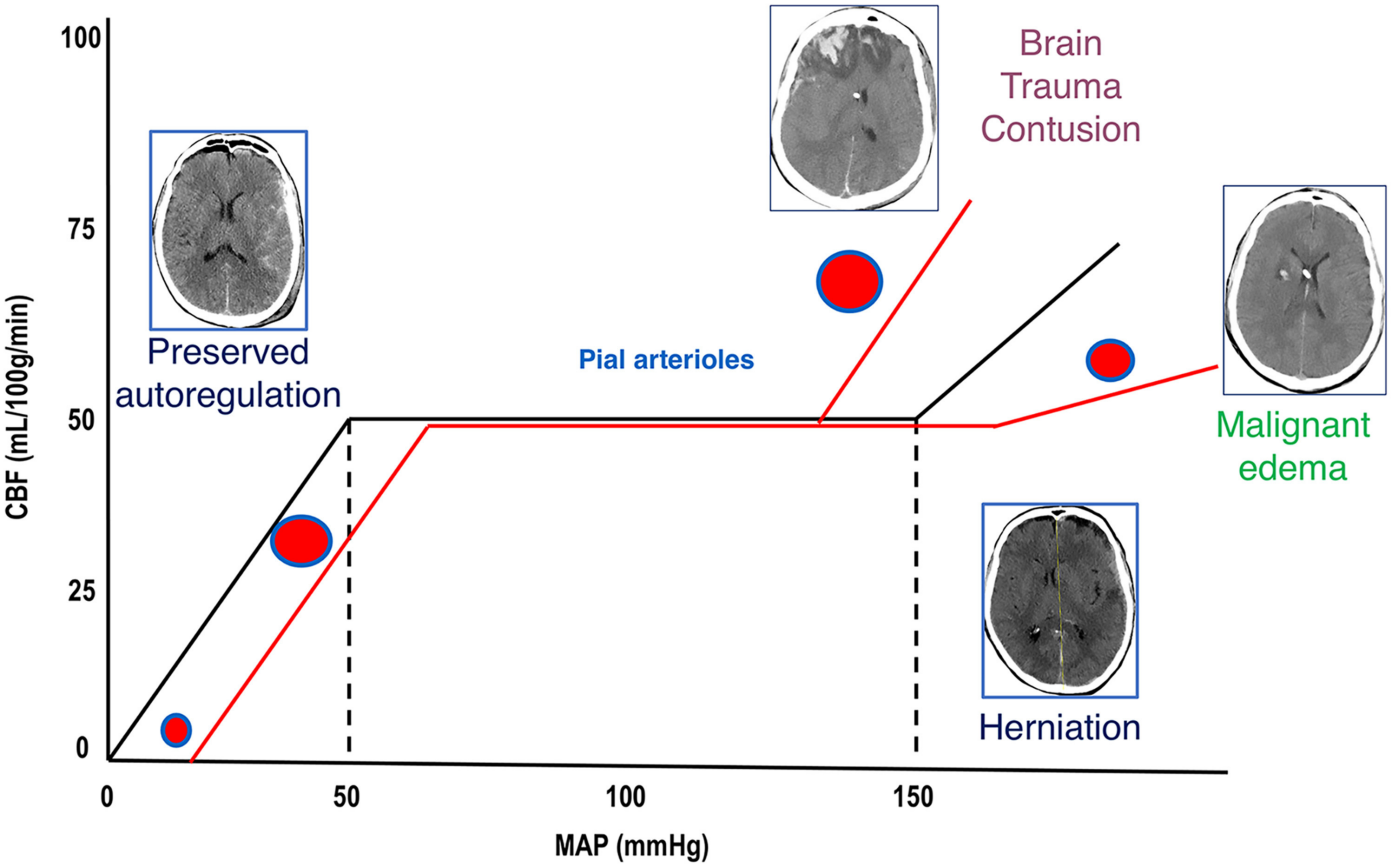
Cerebral autoregulation. Cerebral autoregulation in healthy people is reached at a MAP of 50–150 mmHg and ICP below 20–22 mmHg. After TBI, autoregulation is initially preserved, and compensatory mechanisms act to control ICP and to perfuse the brain (CT scan on the left). When these mechanisms are saturated, cerebral autoregulation is lost, ICP increases, and CBF reduces; if left untreated, this culminates in the worst-case scenario of cerebral herniation (CT scan on the right side). When autoregulation is preserved, pial arterioles dilate in response to ICP increase in order to maintain adequate CBF. When autoregulation is lost, arterioles constrict or dilate causing further reduction of CBF (ischemia) or unnecessary increase of perfusion (hyperemia and contusion evolution or malignant edema). MAP, mean arterial pressure; ICP, intracranial pressure; TBI, traumatic brain injury; CT, computed tomography; CBF, cerebral blood flow, DAD, diffuse axonal damage.

The normal ICP value in adults is around 15 mmHg, increasing physiologically during cough or sneeze. The skull is a closed and rigid container, whose volume consists of three components: cerebrospinal fluid, blood, and brain parenchyma. Cranial volumes and pressures are normally self-equilibrated and self-regulated, thereby keeping cerebral blood flow (CBF) constant in case of variation in any one of these compartments or additional volume. Under normal conditions, the compliance curve that describes the relationship between ICP and intracranial volume is exponential. In the first part of the curve, ICP increases slowly, then rises steeply when the compensatory systems are saturated (as in the case of CSF displacement through the foramen magnum, compression of the cerebral venous system, displacement of brain tissue, and herniation syndromes) (3, 8, 9). After TBI, these mechanisms occur in case of an ICP increase and progressive neurological deterioration. ICP values over 20 mmHg (2, 3, 13) or 22 mmHg (4) are considered pathological in adults, and should follow a conservative “staircase approach” or the surgical evacuation of any hematoma if present (3), with the goal of achieving CPP values between 60 and 70 mmHg (4). Any rise in ICP leads to CPP reduction; indeed, CPP is calculated as the mean arterial pressure minus ICP. CBF impairment may progress until the onset of inadequate oxygenation and ischemia (secondary brain injury), which can lead to cytotoxic edema, resulting in further increase in ICP (2–4, 9, 10). Brain trauma or metabolic impairment can cause tissue ischemia, leading to failure of the sodium-potassium pump with subsequent water influx into the cells, followed by brain swelling and lysis. Other compensatory mechanisms are activated after TBI, such as the sympathetic nervous system, which increases cardiac output and blood pressure and triggers systemic vasoconstriction (14). An overview of ICP pathophysiology is depicted in Figure 1.

## Intracranial Hypertension (HICP): How to Escalate Therapy

The standard management of intracranial hypertension after TBI includes an escalation of therapies, that consists of gradual steps of intervention, which could be skipped when felt indicated; hence, it is not always fundamental to ascend all the steps prior to advancing (i.e., early decompressive craniectomy in selected cases), except for hyperosmolar drugs. While the indication for initiating HICP treatment is clear in case of inv-ICP monitoring (ICP > 20–22 mmHg), the clinical examination and CT-based approach for non-invasively monitored patients is less straightforward. Based on clinical and radiological findings, escalation of therapy should be considered in case of neuroworsening, no improvement or impairment on CT scan, or no response to initial therapy (6). Neuroworsening is defined as a decrease in GCS motor score > 2, loss of pupillary reactivity, new pupillary asymmetry, and/or deterioration of neurological status (6).

The “staircase approach” usually starts from basic advisory (tier zero), till the need for the most aggressive treatments (tier one to three) (15). Stepping from a “baseline” to a higher tier is a potential indicator of increased severity. The higher the tier—the higher the risk, thus in case of non inv-ICP monitoring and neuroworsening, transferring the patient to a tertiary care hospital with more resources is highly recommended (6).

### Tier Zero

Tier “zero” denotes those basic interventions that should be implemented irrespective of ICP elevation, and that can be pursued in all sub-populations of neurocritical care patients. Although no clear consensus has been reached as to which interventions compose this toolset, they include ICU admission, endotracheal intubation and mechanical ventilation, serial neurological evaluation, head-up position (15–30°), analgesia for pain management, sedation to prevent ventilator-patient asynchronies, normothermia, central line placement, end-tidal-CO_2_ monitoring, a CPP threshold of 60 mmHg, hemoglobin > 7 g/dL, normal values of serum sodium, an arterial line for invasive continuous pressure monitoring, and a peripheral oxygen saturation (SpO_2_) ≥ 94% (8, 9).

### Tier One to Three

**Figure 2:**
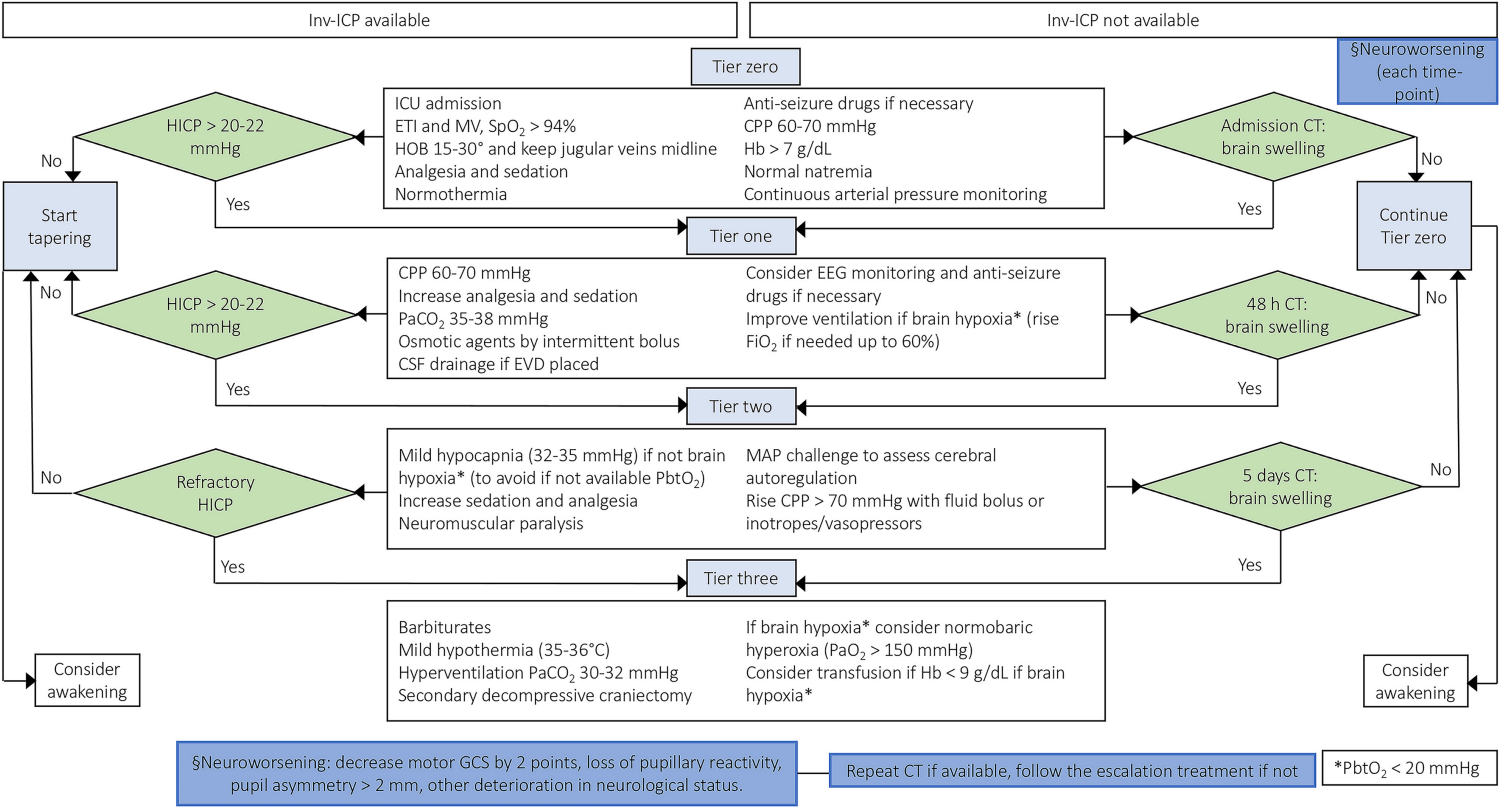
Escalation management for controlling ICP in TBI patients with or without inv-ICP monitoring. Escalation of care in patients with HICP or neuroworsening/radiological impairment [Modified from Hawryluk et al. (9) and Carney et al. (4)]. inv-ICP, invasive intracranial pressure; ETI, endotracheal intubation; MV, mechanical ventilation; CPP, cerebral perfusion pressure; Hb, hemoglobin; PaCO_2_, partial pressure of carbon dioxide; CSF, cerebral spinal fluid; SpO_2_, peripheral saturation of oxygen; HOB, head of the bed; CT, computed tomography; HICP, intracranial hypertension; PbtO_2_, brain tissue oxygen tension; EVD, external ventricular drainage; EEG, electroencephalography; FiO_2_, fraction of inspired oxygen; MAP, mean arterial pressure; PaO_2_, partial pressure of oxygen; GCS, Glasgow coma scale.

Tiers one to three comprise those interventions initiated only in case of HICP: (1) CPP maintenance (between 60 and 70 mmHg) (4), increasing analgesia and sedation, intermittent bolus administration of osmotic agents, cerebrospinal fluid (CSF) drainage if an external ventricular drainage (EVD) device has been placed, partial pressure of carbon dioxide (PaCO_2_) between 35 and 38 mmHg, electroencephalography (EEG) monitoring, and prophylactic anticonvulsants if risk is deemed high; (2) mild hypocapnia (32–35 mmHg), neuromuscular paralysis, mean arterial pressure (MAP) challenge to assess autoregulation using inotropes/vasopressors, and use of inotropes/vasopressors when necessary if autoregulation is intact; (3) barbiturate coma, mild hypothermia (35–36°C), hyperventilation with a goal of 30–32 mmHg PaCO_2_, and secondary decompressive craniectomy (4, 8, 9). These treatments may be implemented with (1) further increase of fraction of inspired oxygen (FiO_2_) up to 60%, (2) ventilator management to reach a partial pressure of oxygen (PaO_2_) up to 150 mmHg, CPP above 70 mmHg, and (3) transfusion of red blood cells if hemoglobin < 9 g/dL to increase the oxygen delivery in case of HICP with hypoxic brain (if brain tissue oxygen tension (PbtO_2_) measurement is available), taking care to avoid moderate-severe hyperventilation in these specific cases (4, 8, 9), Figure 2.

#### Non-Barbiturate Sedatives and Analgesics

Analgesics and sedatives carry the risk of hypotension, which might reduce CPP and increase the risk of brain ischemia. After TBI, it is essential that cerebral oxygen delivery be increased, and cerebral metabolic demand be attenuated to achieve an adequate energy balance and oxygen availability. Sedatives and analgesics are used to suppress metabolism, reduce oxygen consumption and CBF, and improve ICP control (metabolic coupling) (16). Since the main problem of HICP is the decrease in CBF and tissue perfusion, the metabolic effect of sedatives on oxygen consumption becomes marginal (15). The metabolic suppression of cerebral metabolic rate of oxygen (CMRO_2_) induced by sedatives is dose dependent. In particular, CBF reduction should be considered as an adaptive mechanism to reduce brain metabolism, which is dose-dependently suppressed by all intravenous sedative agents (17). Sedatives can exert hemodynamic side effects such as myocardial depression, MAP decrease, and peripheral vasodilatation. These effects should be carefully monitored in patients with impaired cerebral autoregulation, in order to avoid a critical reduction in CPP and oxygen delivery to the brain with possible secondary brain ischemia (18). Otherwise, in patients with preserved autoregulation, the use of sedatives with MAP reduction and compensatory vasodilatation may increase ICP (17). Thus, the use of sedatives and analgesics is essential to protect the brain in the acute phase (within 48 h of injury), and to control HICP.

##### How to Use Sedatives and Analgesics

Suggested sedatives and analgesics for protecting the brain within the first 48 h after TBI (in case of no ICP elevation) include propofol, followed by midazolam, and fentanyl, followed by morphine (17). The use of deeper sedation in mechanically ventilated general ICU patients has been associated with worse outcomes, while in the neuro ICU, it reduces the ability to assess a neurological response (15). The ideal sedative in TBI patients would be able to reduce the CMRO_2_, while maintaining CBF/CMRO_2_ coupling, CPP, cerebral autoregulation, and not raising ICP. Sedatives with antiepileptic and short-term activity should be preferred (19). Moreover, sedation and analgesia should reduce pain and agitation, improve tolerance of the endotracheal tube, and prevent high intrathoracic pressures (e.g., cough) in order to maintain normal ICP values (17).

In presence of HICP, propofol, fentanyl, and rocuronium are used in more than 80% of cases, while midazolam and ketamine are less frequently used (20). Propofol and midazolam seem effective for ICP control (21), although propofol shows greater effects on brain metabolism. These effects are dose-dependent: at <4 mg/kg/h, propofol ensures CBF/CMRO_2_ coupling, adequate brain oxygenation, and cerebrovascular reactivity, while at higher doses (>5 mg/kg/h) it can cause burst suppression (17). Propofol doses can be increased if the EEG monitoring does not suggest metabolism suppression and ICP control is not achieved (16).

Midazolam is supplied as a high-lipid formulation that may cause tissue accumulation irrespective of its short half-life, thus prolonging the weaning phase. Although controversial, midazolam can be suggested over propofol in case of hemodynamic instability (22, 23), but the need for higher doses for ICP control could lead to accumulation, leading to prolonged coma, mechanical ventilation, and ICU length of stay (21).

Ketamine has been avoided for many years to control ICP; however, when compared with opioids, it does not increase ICP and provides an optimal hemodynamic stability, reducing the need for vasopressors (20). Nevertheless, given the limited evidence and persistent doubts concerning its effect on ICP, ketamine is not included among the first-line sedatives for ICP control (24). Ketamine alone is not suggested for ICP management, but it may be administrated (dosage 1–5 mg/kg/h) together with other sedatives to reduce their doses (20).

In summary, a classical protocol for analgesia-sedation in patients with HICP may include propofol 4–6 mg/kg/h and fentanyl 1–4 μg/kg/h, plus vasopressors as needed for the maintenance of CPP at acceptable levels (15). Maintenance of the intravascular volume is mandatory to avoid hypotension during deep sedation (3, 4, 8, 13).

#### Hyperosmolar Therapy

Bolus administration of hyperosmolar therapy represents a fundamental step of the “staircase approach,” which acts by inducing a gradient between the vascular circuit and the brain, determining free passage of water across the blood-brain barrier (BBB) with ICP reduction. Continuous infusion of hyperosmolar drugs is not recommended (25). Three main paths for evacuation of excess fluid from acute cerebral edema have been identified in animal models: (1) via the glia limitans externa to the subarachnoid space, (2) via the glia limitans interna and ependyma to the ventricles/central canal, and (3) via the BBB into the lumen of blood vessels (26). Fluid is also lost into the site of injury, which is converted into a “cavity of injury” (27). Recent research has confirmed that excess edema fluid leaves the brain through an integrated system of astrocytes which overexpress acquaporin-4 (AQP4) (28–30). After infusion of hyperosmolar therapies, plasma volume expansion, higher viscosity, and reduction in CBV are observed. These effects may last for hours, until the normal osmolar gradient is restored. Two medications are currently recommended as first-tier therapies for lowering ICP via osmotic mechanisms: hypertonic saline and mannitol (17).

Mannitol is a mannose sugar alcohol. In addition to moving free water from brain tissue to the interstitial space and vascular compartment, it modifies the osmolarity of glomerular filtrate because it is not reabsorbed by the renal tubules, thereby inhibiting sodium and chloride reabsorption and increasing diuresis (31, 32). However, mannitol has been implicated in the occurrence of renal tubular epithelial damage and acute renal failure, especially in patients with hypo- or normonatremic hyperosmolality. For this reason, both mannitol and hypertonic saline should only be used in patients with normal/low plasma osmolarity, with a target of 300–320 mOsm/Kg. Other negative effects of mannitol include prolonged QTc interval, arrhythmias, and myocardial ischemia. Therefore, many clinicians prefer hypertonic saline over mannitol (9). Hypertonic saline can be used at different concentrations of sodium chloride, each yielding distinct responses (Table 1). Experimental studies have found that hypertonic saline also reduce proinflammatory cytokine levels in activated microglia (66).

**Table 1:** State of the literature concerning mannitol and hypertonic saline for intracranial hypertension.

**Year**	**Study design**	**HTS or M concentration**	**HTS or M dose**	**Effects**
Jagannatha et al. (33)	Randomized controlled trial	HTS 3% M 20%	2.5 mL/kg 2.5 mL/kg	At equimolar doses, HTS and M are equally effective in reducing HICP, but HTS acts faster
Mangat et al. (34)	Retrospective	HTS 3–23.4% and M 20%	NR	HTS reduces HICP more than M, and is less expensive for prolonged ICU stays
Major et al. (35)	Prospective observational	HTS 30%	10 mL	Highly concentrated HTS does not affect laboratory values
Colton et al. (36)	Retrospective	HTS 3%	250–500 mL	When HTS reduces ICP for more than 2 h, it is associated with decreased mortality and long-term disability
Dias et al. (37)	Prospective observational	HTS 20%	0.5 mL/kg	HTS reduces ICP, improves CBF and CPP, and does not affect cerebral oxygenation
Ichai et al. (38)	Randomized controlled trial	Sodium Lactate Isotonic Saline	0.5 mL/kg/h 0.5 mL/kg/h	Hyperosmolar lactate is effective in reducing HICP without modifying plasma osmolarity
Roquilly et al. (39)	Randomized controlled trial	Balanced isotonic Isotonic saline	30 mL/kg/day 30 mL/kg/day	No effects on HICP
Eskandari et al. (40)	Prospective observational	HTS 14.6%	40 mL	HTS administrated as repeated boluses reduces ICP, even in refractory HICP
Diringer et al. (41)	Prospective observational	HTS 20%	1 mg/kg	Mannitol reduces HICP, but does not reduce CBV
Wells et al. (42)	Retrospective	HTS 3 or 7%	150 mL bolus, continuous infusion	Patients with low serum Na+ require more HTS than those with normal serum Na+
Scalfani et al. (43)	Prospective observational	HTS 23.4% M 20%	0.686 mL/kg 1 g/kg	HTS and M reduce HICP, increase CPP, and increase CBF
Paredes-Andrade et al. (44)	Retrospective	HTS 23.4%	30 mL	Boluses of HTS can reduce HICP without modifying serum or CSF osmolarity
Sakellaridis et al. (45)	Randomized controlled trial	HTS 15% M 20%	0.42 mL/kg 2 mL/kg	HTS and M are equally effective in reducing HICP
Roquilly et al. (39)	Retrospective	HTS 20%	Continuous infusion	HTS continuous infusion does not cause HICP rebound when stopped
Bourdeaux et al. (46)	Randomized controlled trial	HTS 5% Na^+^HCO3^−^ 8.4%	100 mL 85 mL	HTS and Na^+^HCO3^−^ are equally effective in reducing HICP
Rhind et al. (47)	Randomized controlled trial	HTS 7.5% IS 0.9%	250 mL 250 mL	HTS reduces neuroinflammation and hypercoagulation
Oddo et al. (48)	Prospective observational	HTS 7.5% M 25%	250 mL 0.75 g/kg	HTS is an effective treatment for refractory HICP to M, also improving CPP
Kerwin et al. (49)	Retrospective	HTS 23.4%M	30 mL	HTS and M are equally effective in reducing HICP
Ichai et al. (50)	Randomized controlled trial	Sodium Lactate M 20%	1.5 mL/kg 1.5 mL/kg	Hyperosmolar lactate is effective in reducing HICP and the effect is maintained longer than M
Froelich et al. (51)	Retrospective analysis of prospective data	HTS 3%	1.5 mL/kg bolus, continuous infusion	HTS can cause hypernatremia and induce renal dysfunction (especially when serum Na+ >155 mEq/L)
Rockswold et al. (52)	Retrospective	HTS 23.4%	30 mL	HTS reduces HICP and increases CPP
Francony et al. (53)	Randomized controlled trial	HTS 7.45% M 20%	100 mL 231 mL	M and HTS are equally effective in reducing HICP. HTS is preferred in hypovolemic and hyponatremic patients; M is preferred in hypoperfused patients
Sorani et al. (54)	Retrospective	M 20%	50–100 g	Each 0.1 g/kg increase in M decreases ICP by 1 mmHg, only in case of HICP
Sakowitz et al. (55)	Prospective observational	M 20%	0.5 g/kg	M reduces HICP by tissue dehydration
Soustiel et al. (56)	Prospective observational	M 20%	0.5 g/kg	M reduces HICP and increases CPP as hyperventilation does. CBF improves with M in respect to hyperventilation
Ware et al. (57)	Retrospective	HTS 23.4% M 75 g or 0.86 g/kg	continuous infusion bolus	HTS and M are equally effective in reducing HICP. HTS acts longer than M
Gasco et al. (58)	Prospective observational	M 20%	100 mL	M reduces HICP and improves cerebral oxygenation
Munar et al. (59)	Prospective observational	HTS 7.2%	1.5 mL/kg	HTS reduces HICP without affecting hemodynamics for at least 2 h
Horn et al. (60)	Prospective observational	HTS 7.5%	2 mL/kg	HTS can reduce HICP even in cases refractory to mannitol
Suarez et al. (61)	Retrospective	HTS 23.4%	30 mL	HTS reduces HICP and increases CPP
Hartl et al. (62)	Prospective observational	M 20%	125 mL	M reduces HICP, increases CPP, and does not alter cerebral oxygenation
Hartl et al. (63)	Prospective observational	HTS 7.5%	Continuous infusion	HTS reduces HICP, increases CPP, and does not affect hemodynamics
Unterberg et al. (64)	Prospective observational	M 20%	125 mL	M reduces HICP. If CPP>60 mmHg, M does not improve brain tissue oxygenation
Fortune et al. (65)	Prospective observational	M	25 g	M reduces HICP, but increases CBV

*M, mannitol; HTS, hypertonic saline; ICP, intracranial pressure; HICP, intracranial hypertension; CPP, cerebral perfusion pressure; CBF, cerebral blood flow; CBV, cerebral blood volume*.

##### How to Use Osmotic Agents

There is still no consensus as to which osmotic agent is superior for controlling ICP without major side effects. Mannitol is commonly administered at the dose of 0.25–1 g/kg every 4–6 h, while the concentration of hypertonic saline can vary from 3 to 7% and even 23.4% (Table 1). Their effects continue for 4–6 h until the normal osmolar gradient is restored. They also lead to hemodilution, as well as increased cardiac output and blood pressure (15). Possible adverse effects of mannitol include dehydration, hypovolemia, and renal damage, whereas hypertonic saline may lead to dangerous hypernatremia (3, 25). In fact, if severe hypernatremia develops rapidly, it could causes shrinking of the brain with vascular damage and subsequent hemorrhage. Acute hypernatremia could also lead to central nervous system demyelination, while chronic hypernatremia may lead to encephalopathy (67). Although current guidelines for the management of severe TBI suggest the use of mannitol (0.25–1 g/kg body weight) over hypertonic saline for HICP control, the debate between these two approaches is still open (3, 4, 25, 68). Table 1 summarizes all studies available in literature from 1995 to 2020 concerning osmotic therapies for the treatment of HICP. In this line, recent studies confirmed that mannitol is not superior to hypertonic saline in terms of long-term efficacy and safety after TBI (68–70). A useful strategy is to test both agents with an equimolar bolus, in order to evaluate which therapy has the greatest efficacy for each patient (15).

Limited, retrospective data on continuous infusion of hypertonic saline suggest that patients with low serum sodium require more hypertonic fluid than those with normal serum sodium, while those with serum sodium >155 mEq/L can develop hypernatremia and renal dysfunction. Moreover, continuous infusion of hypertonic saline does not cause rebound HICP when stopped and has demonstrated equal efficacy in reducing HICP than mannitol, increasing CPP without affecting hemodynamics (Table 1). Therefore, the potential efficacy of a continuous infusion over bolus may be related to the patient's osmolarity, but further studies are needed to corroborate this hypothesis. In the absence of more conclusive evidence, hyperosmolar therapies (whether hypertonic saline or mannitol) should be administered by bolus; continuous infusion is not recommended (25).

#### Cerebral Spinal Fluid (CSF) Drainage

In case of inv-ICP monitoring with an external ventricular drainage (EVD) system, CSF drainage represent an effective technique to reduce ICP, but there is no strong evidence of ICP long-term reduction (3, 4). Intraventricular ICP monitoring device consists of a catheter with a transducer (fiberoptic strain gauge or pneumatic sensor) placed into the cerebral ventricle system, which is connected to an external pressure monitoring system capable of ICP detection (71). It is an EVD, which allows CSF subtraction in case of HICP. Intraventricular ICP is the first and oldest system of inv-ICP monitoring described (72, 73), and still represents the more reliable device capable to detect ICP and to assess intracranial compliance (71, 74, 75). The device is usually placed in the frontal horn of the ventricle through the Kocher point, 2 cm anteriorly to the coronal suture and 2.5–3 cm laterally from the midline, directed toward the intersection point between the sagittal plane on the medial canthus of the ipsilateral eye and the coronal plane on the external auditory meatus (approximately the location of the foramen of Monro) (76, 77). The most correct calibration point (zero-point) should be at the foramen of Monro (at level of the external auditory canal). Complications associated to the placement of intraventricular inv-ICP device may include technical problems misplacement, dislocation, kinks, obstruction from debris, and blood (74, 78–80), which ranges from 4.5 to 25% (81–83), hemorrhage [reported in 0.7 and 0.61% two meta-analysis considering only the symptomatic bleeding (80, 84) and in 2.5% (considering all hemorrhages) (81), and infection [which ranges from 1 to 27%, and is correlated with the duration of device maintenance and number of tapping (85)]. In short, intraventricular inv-ICP represents the best device for intracranial compliance evaluation, but it needs a careful management due to possible complications. Furthermore, intraventricular device is often difficult to position in young patients because of the smaller ventricle volume, and in TBI patients with HICP in whom the ventricular system is collapsed as a compensatory mechanism (75). Although ventricular inv-ICP monitoring is usually considered the “gold standard,” variable impacts on long-term outcome have been shown in studies comparing intraventricular and intraparenchymal systems (7, 86–88). We therefore recommend either placement of an inv-ICP device when indicated, or monitoring of the ICP by non-invasive means (i.e., transcranial doppler, optic nerve sheath diameter) (89).

#### Partial Pressure of Carbon Dioxide Management

Cerebral physiology is deeply modified by PaCO_2_ changes. PaCO_2_ can modulate vasomotor tone, leading to cerebral vasoconstriction in case of hypocapnia, or cerebral vasodilatation in case of hypercapnia (90, 91). A systematic review demonstrated that both hypocapnia and hypercapnia are associated with poor outcomes after TBI (92). Hypocapnia can reduce CBF and cerebral blood volume (CBV) and is usually achieved through hyperventilation. Hyperventilation decreases ICP and induces brain relaxation. Despite the well-established efficacy of hyperventilation for ICP control, the effect of this practice on long-term outcome is unclear. Hypocapnia may increase cerebral metabolic activity by raising oxygen and glucose consumption, producing excitatory amino acids, and triggering the switch to anaerobic metabolism, thereby increasing the risk of seizures and hyperexcitability. Patients with TBI show less CBF reduction than those with uninjured brains, due to the fact that hyperventilation redistributes blood flow to injured tissue. Finally, hyperventilation followed by hypocapnia may lead to alkalosis by shifting the oxygen-hemoglobin dissociation curve (Bohr effect) (91, 93).

##### Induction of Hypocapnia

Hyperventilation can be performed by increasing tidal volume or respiratory rate in mechanically ventilated patients (4, 94). In general neurocritical care as part of the “tier zero,” PaCO_2_ should be maintained between 35 and 38 mmHg, while prophylactic moderate hyperventilation in case of HICP should be weighted on risk/benefit to patients, considering that it may be harmful for GCS < 4–5 (62), because PaCO_2_ levels between 20 and 25 mmHg correspond to a 40–50% decrease in CBF (90). Particularly, literature on pre-hospital TBI cares suggests to avoid hyperventilation within the first 24 h following TBI, except in clear case of refractory HICP or cerebral herniation (4). Besides, in case of elevated ICP mild hypocapnia (32–35 mmHg) could be considered. On the other side, a brief period (15–30 min) of hyperventilation in case of refractory HICP, targeting PaCO_2_ of 30–32/30–35 mmHg (for the SIBICC/BTF guidelines, respectively), may be appropriate. However, prolonged hypocapnia should be prevented (4, 8, 9). Hyperventilation is not devoid of complications. In fact, brain ischemia may represent a potential harmful side effect of this treatment. In a randomized controlled trial in which patients were randomized to receive normal ventilation (PaCO_2_ 35 mmHg), moderate hyperventilation (PaCO_2_ 25 mmHg), or tromethamine (THAM) plus hyperventilation, hyperventilation for 5 days resulted in worse outcomes at 3–6 months. Better 12-month outcomes were found in the THAM plus hyperventilation group (95). This was also confirmed by Brandi et al. (96), who showed that 50 min of hyperventilation do not change glucose, lactate, or pyruvate concentrations, but can modify brain tissue oxygenation tension. Hence, considering patients with HICP and brain hypoxic damage, the SIBICC consensus does not suggest hyperventilation (8, 9). As early as 1997, a Cochrane review found that data were inadequate to conclude whether hyperventilation could be considered detrimental or beneficial for the treatment of acute TBI; in 2008, an updated review reached the same deduction (97). Notwithstanding these conclusions, hyperventilation, is effective for HICP therapy in non-hypoxic brain. However, since the PbtO_2_ monitoring is occasionally available and the hypoxic brain (PbtO_2_ < 20 mmHg) difficult to detect without such specific monitoring, hyperventilation should be used as a last resort.

#### Metabolic Suppression Management (Barbiturates)

Barbiturates are gamma-aminobutyric acid (GABA) receptor agonists which suppress cerebral electrical activity, leading to a reduction in CBF, CPP, and CBV. The reduction in CBF is proportional to the CMRO_2_ and lowers ICP. Barbiturate therapy was widely employed for decades in the management of TBI patients refractory to “second-tier” interventions (94), given the ability of these agents to suppress brainstem reflexes, cerebral activity and metabolic demand, until potentially reaching the deepest state known as burst suppression (98). The aim of barbiturates administration is to control ICP, and their effects on cerebral metabolism should be observed through EEG monitoring. A state of burst suppression is not the goal of barbiturates, and if it appears, no further dose increases are indicated (9, 94, 99). Barbiturates also induce vasoconstriction and decrease cardiac output, thus modulating cerebral metabolic demand, with no effects on mortality or disability. Barbiturates are indicated only for the treatment of refractory HICP and refractory seizures, and should be titrated to the lowest effective dose (17). EEG should be used to guide titration of therapy, as it is now known that burst suppression is not the aim of barbiturate administration and must not be pursued if ICP control has been obtained. Likewise, increasing barbiturate doses in case of refractory HICP should be avoided if burst suppression is already present, as it is unlikely to lead to further reduction of ICP (9, 94, 99). One-fourth of patients treated with barbiturates can develop hypotension, which mirrors the substantial effects of CPP on ICP (100). Other complications include respiratory depression, infections, immunosuppression, hepatic, and renal dysfunction (101).

##### Induction of Metabolic Suppression

Initial therapy with barbiturates consists of a bolus followed by continuous intravenous infusion for maintenance (14). Thiopental and pentobarbital are the most used barbiturates. Thiopental is metabolized into five metabolites, one of which is pentobarbital (102); this may explain the higher efficacy of thiopental when compared to pentobarbital. When compared to thiopental, pentobarbital is less effective in reducing ICP as first-line therapy (102). The classic dose of pentobarbital should be 5–7 to 10 mg/kg, while thiopental should be used with a median loading dose of 15 mg/kg followed by continuous infusion of 100 mg/kg/day (99). Depressive effects on the central nervous system occur within 15 min, but this varies from patient to patient.

#### Decompressive Craniectomy (DC)

Decompressive craniectomy (DC) consists in the removal of a portion of skull in order to treat refractory HICP and represents the most aggressive step of the “staircase approach” (103). When the bone flap is not replaced after surgery for the evacuation of an intracranial mass lesion, DC is named “primary,” while it is considered “secondary” when DC is performed later after other treatments have failed (104). DC can be performed as a large frontal-temporal-parietal flap (at least 12 × 15 cm diameter) (104) or as a bifrontal flap; both techniques have shown an efficacy close to 100% for ICP control (1–4, 9, 13, 99, 105). However, the optimal indications, technical aspects, and timing for DC are still debated. Two major multicenter randomized controlled trials (RCTs) comparing decompressive craniectomy with medical management tried to provide guidance to clarify timing and indications of DC: Decompressive Craniectomy in Patients with Severe Traumatic brain Injury (DECRA) and Trial of Decompressive Craniectomy for Traumatic Intracranial Hypertension (RESCUEicp) (2, 13). The DECRA trial showed a similar rate of mortality between medical and surgical cohorts, with a higher rate of unfavorable neurologic outcomes in the surgical group. On the other hand, the RESCUEicp study observed lower mortality for DC, but higher rates of vegetative state, as well as lower and upper severe disability at 6 months, in comparison to medical therapy (2). A key difference between the two studies was that DECRA investigated the effects of DC for *early* HICP, while the effects of DC for *late* HICP were analyzed by RESCUEicp (104). In fact, DECRA included patients with HICP (> 20 mmHg) for 15 min over a 1-h period although the tier 1 therapies within the first 72 h after trauma, while RESCUEicp included patients with HICP (> 25 mmHg) for 1 to 12 h despite the tiers 1 and 2 therapies within the first 10 days after TBI (2, 13). Therefore, the interpretations and recommendations extrapolated from these studies should refer to *early* and *late* refractory HICP. A recent update on DC by the Brain Trauma Foundation (104), based on the RESCUEicp and DECRA findings, developed Level IIA recommendations, suggesting that secondary DC for *early* refractory HICP is not recommended to improve mortality and outcome, while is suggested in case of *late* refractory HICP. Otherwise, DC performed both in *early* and *late* refractory HICP is recommended to reduce ICP and ICU length-of-stay. Moreover, Authors observed that bifrontal DC (the technique used in the DECRA trial) is effective to reduce ICP and ICU-stay, but it is not recommended to improve outcome and mortality if performed in accordance with the DECRA inclusion criteria. Besides, the 2020 update of the BTF guidelines (104) concluded that a frontal-temporal-parietal DC (12 × 15 cm) is recommended over a small flap for mortality and outcome improvement after severe TBI (106, 107). Many other studies analyzed the use of DC in severe TBI and its implications for long-term neurological outcome, confirming its efficacy for ICP control and reduction of mortality, but increasing long-term disability (108–115). The socioeconomic context, patients' priorities, and the recognition of clinical and radiological prognostic factors (for which further validation studies are needed) should be considered before indicating DC.

## Tapering Therapies After the Control of Intracranial Hypertension

**Figure 3:**
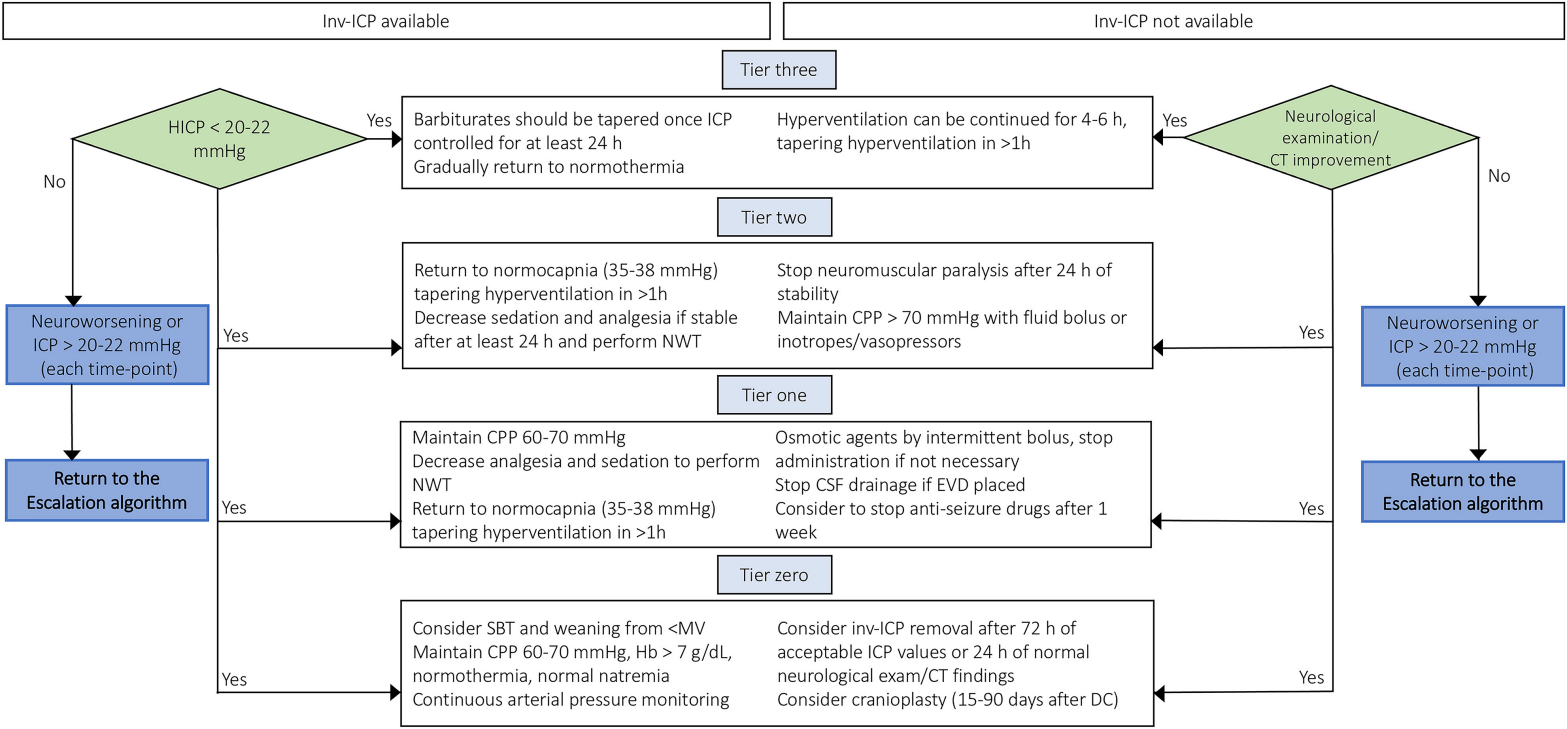
De-escalation management after controlling intracranial hypertension in TBI patients with or without inv-ICP monitoring. De-escalation management for controlling intracranial hypertension basing on available current evidences [Modified from Stocchetti et al. (3), Hawryluk et al. (9) and Carney et al. (4)]. inv-ICP, invasive intracranial pressure; CPP, cerebral perfusion pressure; Hb, hemoglobin; PaCO_2_, partial pressure of carbon dioxide; CSF, cerebral spinal fluid; CT, computed tomography; HICP, intracranial hypertension; EVD, external ventricular drainage; NWT, neurological wake-up test.

Once HICP is controlled, the aggressive therapies applied following the “staircase approach” should be carefully tapered in order to avoid secondary brain damage induced by excessive brain metabolism suppression, reduced oxygen delivery, and impaired systemic hemodynamics with dangerous consequences to the brain. Based on the aforementioned, the choice to taper therapies should be weighted on the stability of ICP, but it is extremely hard to define in patients who do not present an inv-ICP monitoring. Figure 3 depicts a possible step-by-step approach for the tapering of care after HICP control.

### Timing for Cranioplasty After Decompressive Craniectomy

By definition, decompressive craniectomy creates a skull defect of varying size and complexity. Cranioplasty has the goal of restoring brain protection, CSF dynamics, and aesthesis after DC (116). Although cranioplasty itself is a routine procedure, it still carries a significant complication rate (100, 117), affecting 23.8–26% of patients (118, 119) (range 7–47%) (116). Risk factors for developing complications after cranioplasty include previous surgery, *in-situ* ventriculoperitoneal shunt (VPS), and systemic and cardiovascular comorbidities (118). Wound complications (e.g., dehiscence, ulcers, necrosis) are reported in 1.6% of cases (118), and may be caused by poor preoperative conditions, underlying infection, or inadvertent sacrifice of the skin flap vascular supply during DC (116). Infection is described in 3–12% of cases (116, 118, 120, 121). Hydrocephalus is reported in 10–45% of cases after DC, but resolution of ventriculomegaly after cranioplasty is well-documented; it appears to persist only in 1–5% of cases (118, 121). Epidural or subdural hemorrhage is described in 3–7% of cases, and is more frequent in case of VPS placement, whereas new-onset seizures are reported in 3–8% of cases (118, 119, 121). The optimal timing for cranioplasty after DC is a matter of debate, considering its hypothetical influence on postoperative infection (116, 119). A recent review (116) described that it is usually performed from 4 to 12 weeks after DC, in accordance with the possible scenarios that could influence the timing of cranioplasty: in the first scenario, the brain is depressed with respect to the skull defect because of post-traumatic brain atrophy or VPS *in situ* (high risk of post-cranioplasty blood collection); in the second scenario, the brain is in physiological position at the level of the inner table of the skull; while, in the third and worst scenario, the brain is over the level of the skull defect, because of edema or hydrocephalus (116). This review showed that the infection rate is higher within the first 14 days after DC (116), probably because a recent healing wound represents a weak point in which normal immune-cell recruitment is altered (118). Iaccarino et al. observed a higher incidence of hydrocephalus within the first 90 days, while seizures were more common after 90 days (116). Thus, an early cranioplasty (15–30 days after DC) may reduce the risk of infection and seizure. Archavlis et al. (122) retrospectively observed a better neurological outcome for patients who underwent cranioplasty within 7 weeks and between 7 and 12 weeks when compared to patients whose cranioplasty was performed at > 12 weeks. However, a higher rate of infection in those with comorbidities (such as diabetes, colonization with multidrug-resistant pathogens, and thromboembolism) was found in the early cranioplasty group. Thus, the authors concluded that the indication for early cranioplasty should take into account both the clinical and neurological patients' status, to better define the optimal timing of surgery and minimize the risk of complications (122). Many other studies reported similar conclusions (116): some authors described a lower rate of complications in *early* cranioplasty, while others observed no impact on complication rate. Few studies found that cranioplasty timing can influence the persistence of hydrocephalus and long-term neurological outcome. However, the most recent meta-analysis by Malcolm et al. (123) reported improved neurological function for patients who underwent an earlier cranioplasty (<90 days after DC). In summary, as observed by the most recent studies (116, 123), there is a growing trend to perform earlier cranioplasties (15–90 days after DC), although there is only low-grade evidence (Class IIb, Level C) to support this. The timing of cranioplasty should be based on the neurological, clinical, and infective status of each patient; surgery should be performed as soon as brain swelling, and clinical condition allow intervention with a lower risk for the patient. A randomized controlled trial on the best timing for cranioplasty after DC, sponsored by NIHR Global Health Research Group, is ongoing, and may clarify the optimal management (116).

### Weaning From Metabolic Suppression

Once a normal ICP value is reached, or clinical examination and imaging (in patients without inv-ICP) are improved, discontinuation of barbiturates can be initiated if the medical staff deems appropriate. The infusion should be tapered, not discontinued abruptly (14). When compared to decompressive craniectomy, thiopental (15 mg/kg followed by 100 mg/kg/day) was equally effective for the treatment of refractory HICP (105). The effects of thiopental can take 4 days to be observed (124). To date, there is no consensus on the duration of barbiturate therapy for refractory HICP (94), although some weaning protocols have been proposed (14). In a study performed on 153 TBI patients with HICP, barbiturates were used for a median time of 4 days, with a range of 2–12 days (99). Withdrawal from barbiturate therapy may result in serious issues, including possible rebound HICP and seizure activity. During discontinuation of therapy, both the long half-life of these drugs and their possible interactions must be taken into consideration; constant monitoring of drug levels has been suggested (14). In summary, we suggest tapering the barbiturate dose once ICP has been controlled for at least 24 h and discontinuing administration only if there is no rebound effect on ICP with progressively lower doses.

### Return to Normocapnia

The BTF guidelines and the SIBICC consensus suggest proceeding with a brief period (15–30 min) of hyperventilation, targeting PaCO_2_ levels to 30–35/32–35 mmHg or lower (30–32 mmHg) if more aggressive treatments are needed (4, 8, 9). Mild hyperventilation cannot be continued for long time; after 4–6 h, physiological buffer systems normalize the pH of the perivascular space, limiting the beneficial effects of hypocapnia, increasing CBF, and causing hyperemia with possible rebound of HICP (90). Moreover, hypocapnia may induce deleterious systemic effects, including decreased blood perfusion of the kidneys, gastrointestinal system, skin and skeletal muscles; platelet adhesion and hyper aggregation; bronchoconstriction, reduced hypoxic pulmonary vasoconstriction, surfactant production, and increased permeability of the alveolar-capillary membrane; respiratory alkalosis with potassium, calcium, and phosphate imbalance; and possible increase in coronary metabolic demand, with coronary spasm, myocardial ischemia, and arrhythmic complications (90).

In short, mild to moderate hyperventilation should be considered only in case of uncontrolled HICP at risk for cerebral herniation syndrome, life-threatening HICP elevation, HICP caused by hyperemia, and aggressive “second-tier therapy” for the control of refractory HICP, should be performed for 15–30 min only and should be avoided if there is risk of brain hypoxia. PaCO_2_ and arterial partial pressure of oxygen (PaO_2_) should be strictly monitored by using end-tidal carbon dioxide or serial arterial blood gases. When hyperventilation is initiated, it must not be stopped abruptly due to the risk of rebound HICP; instead, it should be tapered progressively by reducing respiratory rate over 1 h until normal PaCO_2_ values (35–38 mmHg) are achieved (57).

### When to Stop Osmotic Agents

As noted above, the current evidence indicates that both mannitol and hypertonic saline should be administered as on-demand boluses, and strictly guided by ICP values. Once ICP control has been obtained (ICP < 20–22 mmHg), further boluses should be withheld (3, 8, 9, 15).

A retrospective study by Schomer et al. (125) evaluated the role of dexmedetomidine for refractory intracranial hypertension and for de-escalation from hyperosmolar therapies. The authors observed a reduction in the number of hyperosmolar boluses after initiation of dexmedetomidine. The difference was significant for mannitol (*p* = 0.03), but not for hypertonic saline (*p*=0.20). There were no differences in episodes of hypertension, bradycardia, or CPP reduction. The authors concluded that dexmedetomidine could be a useful adjunct in the management of refractory HICP, reducing the need for hyperosmolar fluid without compromising hemodynamics (125). However, since this approach is extremely new and not confirmed by larger studies, the conventional use of hyperosmolar therapy alone is strongly recommended.

### How to Wean From Sedatives and Analgesics

De-escalation of sedatives should not be encouraged during the first phases of ICP management; it is universally accepted that patients who suffer from HICP need sedation for at least 24 h, and sedation should not be discontinued as long as ICP values remain high (15). The decision to discontinue sedation and analgesia once ICP control is achieved is based on clinical neurological examination, optimization of patient status (e.g., maintenance of euvolemia, fluid balance, monitoring of respiratory, and hemodynamic parameters), appropriate levels of CPP (60–70 mmHg, according to the autoregulatory status and using vasopressors if needed), and appropriate mechanical ventilation to maintain normoxia and normocapnia (SpO_2_ > 94% and PaCO_2_ around 35 mmHg) (15). The neurologic wake-up test (NWT), which consists in reducing sedation and analgesia as part of the daily clinical examination, is not mentioned in TBI guidelines, although it is the only available test that could reliably detect neurological deterioration or improvement and focal neurological deficits (126), thus facilitating clinical decision-making. When performing NWT, the patient should be carefully monitored for ICP and CPP and placed in the supine position. Those few studies that have investigated the role of NWT in TBI concluded that it increases ICP and MAP, although there was no evidence of either brain injury exacerbation or benefit of the test (126).

**Figure 4:**
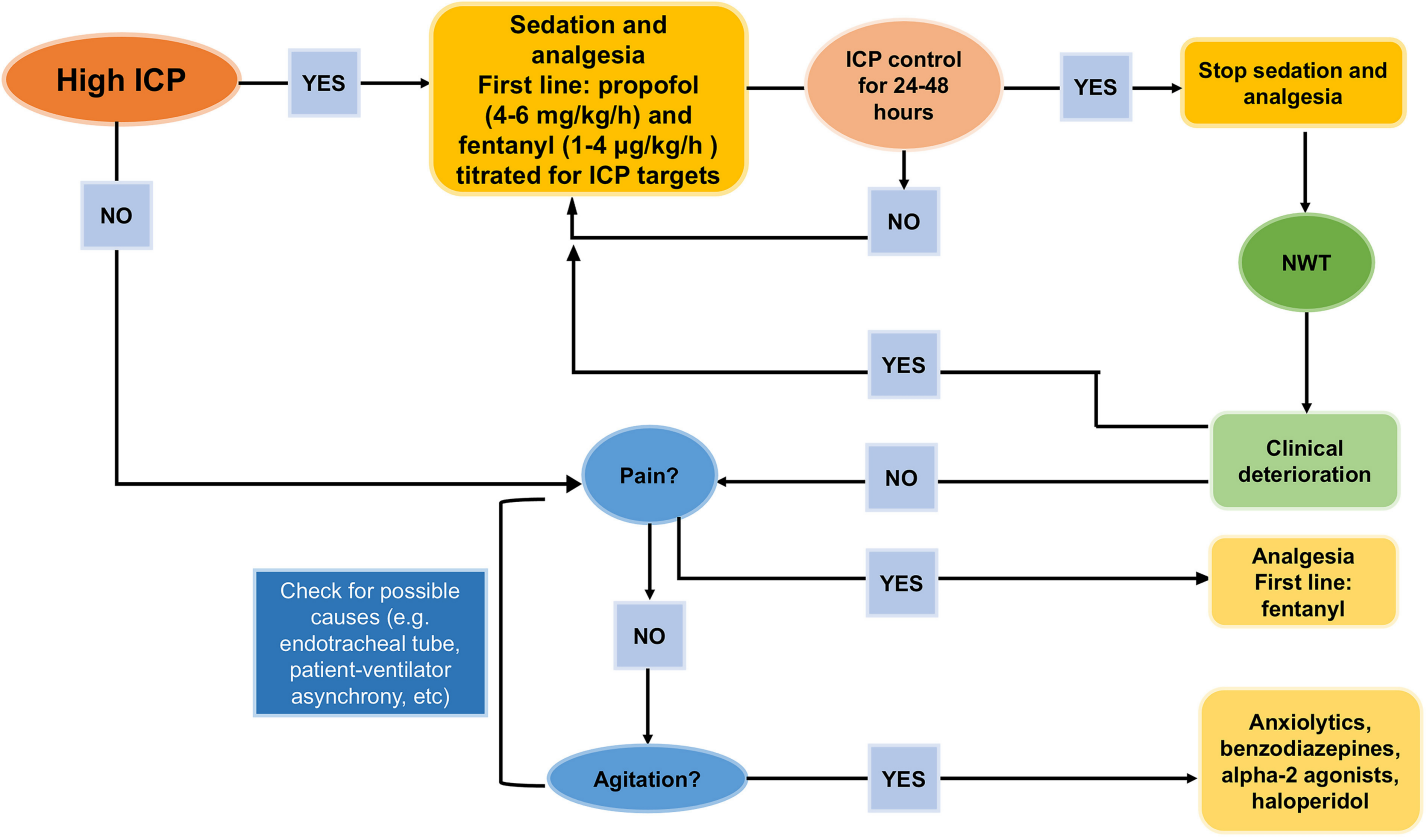
How to de-escalate from sedatives and analgesics. Proposal for de-escalating sedatives and analgesics after intracranial hypertension control [Modified from Oddo et al. (17)].

In patients requiring sedation for longer than 7 days, propofol should be discontinued due to the risk of “propofol infusion syndrome” at doses > 4 mg/kg/h (15, 16, 20). This syndrome is characterized by rhabdomyolysis, green urine, elevated hepatic enzymes, and elevated triglycerides (127). In summary, a combined regimen of propofol (3 mg/kg/h), to reduce oxygen consumption and ensure suppression of seizures, and fentanyl (1–2 μg/kg/h), to facilitate patient-ventilator synchrony, could be recommended. At this dosage, propofol infusion can be withdrawn to allow a neurological examination (16). Propofol and fentanyl should be progressively reduced after at least 24 h of ICP control, except for patients who are still in the acute phase after TBI. In these cases, analgesia and sedation should be continued for 24–48 additional hours to protect the injured brain (17). Before weaning from sedatives and analgesics, endotracheal tube intolerance, and patient-ventilator asynchronies should be excluded as a matter of course (17). Once weaning has begun, the patient's pain and agitation should be carefully evaluated in order to avoid rebound HICP. Dexmedetomidine is a rapidly metabolized alpha-2 agonist that can provide adequate agitation control to allow neurological examination after withdrawal of sedation, but few data are available on its long-term effects in TBI. Figure 4 depicts a proposed algorithm for sedative escalation and de-escalation in case of HICP and thereafter.

### Inv-ICP Monitoring Removal

There is no universal consensus on inv-ICP monitoring in patients who are not neurologically evaluable. The timing of inv-ICP removal remains a matter of debate; the useful information that can potentially be gleaned from its maintenance even after ICP control has been achieved should be balanced with the complications associated with prolonged invasive monitoring (82, 124, 128, 129). Thus, removal of inv-ICP monitoring in these patients should be based on assessment of the risk/benefit ratio, given the potential for complications both as a result of insertion of the inv-ICP probe and of its prolonged maintenance *in situ* (e.g., infection and technical problems) (81, 82, 124, 128–134). The infection rate of inv-ICP monitoring ranges from 1 to 27% (3), and is usually related to the insertion procedure and the duration of monitoring (135). Winfield et al. did not observe a higher occurrence of infections in patients with longer inv-ICP monitoring, and they suggested that weaning from inv-ICP monitoring should be evaluated on a case-by-case basis, considering the true utility of continued monitoring after many days of controlled ICP (132). The SIBICC recommended removal of inv-ICP monitoring after 72 h of acceptable ICP values, and as soon as 24 h for those cases with normal CT-scan findings and who are neurologically evaluable (8).

## Research Agenda

### A Former Point-of-View for Novel Pathophysiological Approaches

**Figure 5:**
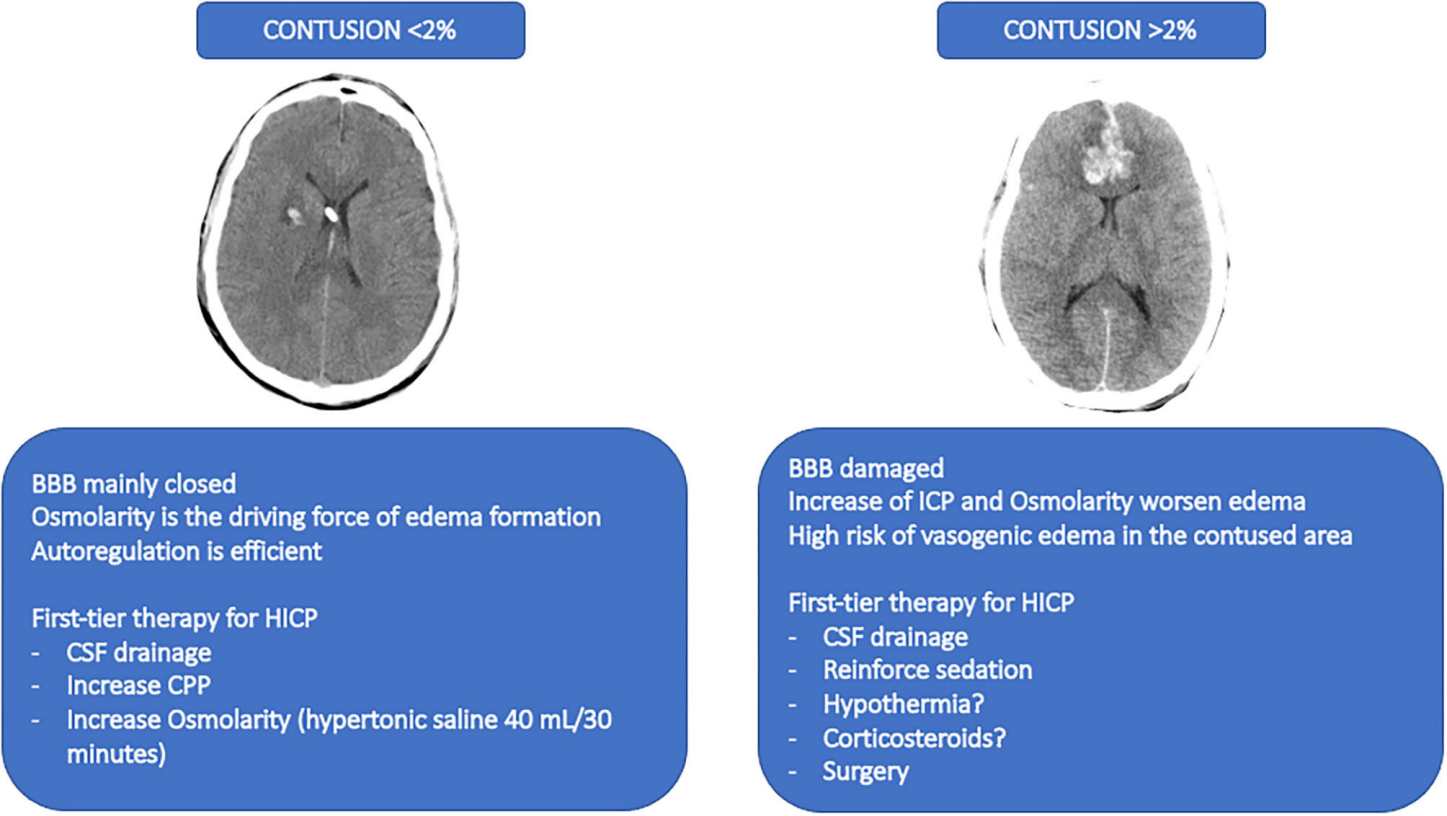
A former point-of-view for novel therapeutic approaches. The figure depicts a proposed therapeutic approach based on a former point-of-view no longer investigated and that should be reinterpreted in light of the progresses in TBI research. The image on the left represents a CT-scan with a contusion <2%. The suggested therapies for this condition are described below. The image on the right represents a CT-scan of a contusion of more than 2%. The suggested therapies are described below. BBB, blood brain barrier; HICP, intracranial hypertension; CSF, cerebral spinal fluid; CPP, cerebral perfusion pressure.

The degree of damage of the BBB is nowadays not considered as individualized therapy after TBI according to the individual pathophysiology. The occurrence of raised ICP in most of the cases is due to cerebral edema. Brain edema can be vasogenic when extravasation of fluid into the extracellular space occurs, followed by BBB damage; while cytotoxic edema appears as a consequence of the passage of extracellular water into the intracellular compartment, mainly due to the ionic gradient (136, 137). The initial hypothesis of BBB damage after TBI includes an acute initial opening of the BBB, followed by the leak of plasma and cells increasing the brain specific gravity with diffuse and homogeneous distribution in the white and gray matters. This mechanism is of short duration and occurs in about 1/4 of the patients with severe TBI, independently of lesions at magnetic resonance images (MRI), thus remaining sequelae for about 2 weeks. It can also worsen the prognosis (138) through cerebral herniation (139, 140) MRI with apparent diffusion coefficient is used to distinguish between vasogenic and cytotoxic edema in TBI patients. While freely diffusible water at MRI is marker of vasogenic edema, restrict water movement represents cytotoxic edema (139). This is associated with a rapid disruption of the BBB within the first hours after the trauma, followed by a biphasic edema formation, starting from the vasogenic, and thus continuing with the cytotoxic until the minimum level after 1 week (141). However, this technique is not easily applied in the first phase of TBI when the patient could be unstable. Unfortunately, CT-scan still not allow the same information as RMI but is considered the first line diagnostic tool in the acute phase of TBI. Besides, volume, weight, and specific gravity can be analyzed. Data from CT images suggested a heavier brain tissue after trauma (136). In this line, a complete destruction of the BBB is associated with leakage of water, proteins and electrolytes with higher density than the brain; while a partial BBB destruction is associated with an added volume characterized by lower density in respect to the brain. In the acute phase of TBI, patients with increased density received more osmotherapy, had more frequently an external ventricular drainage positioned with possible CSF drainage, and received second-tier therapies more often. This suggested that in case of contusion interesting <2% of the brain, the BBB is predominantly intact, the osmolarity is the main driving force for edema formation, and the autoregulation is efficient (increasing pressure decreases cerebral blood volume) (142). In this setting, the first-line treatment of increased ICP could be CSF drainage, increase of CPP, and increase of osmolarity (by using hypertonic saline 40 mL/30 min) (143). The 2020 guidelines for the treatment of cerebral edema recommends *pros* the use of osmotic agents in the hospital setting, but *cons* in the pre-hospital setting (137). Otherwise, if the brain is contused in more than 2% of its tissue, the BBB can be disrupted in a large percentage. The increase of pressure and osmolarity worsens the edema, while the vasogenic edema should be prevented in the contusion area (144). In this setting, the first-line treatment of increased ICP could be the CSF drainage, reinforce of sedation, implement of hypothermia, and corticosteroids (143). This old but also innovative point of view should be further discussed and corroborated, since some of these therapies have been abandoned without trying to distinguish between patients who can benefit and those who cannot. This concept is proposed in Figure 5.

## Conclusions

The stepwise approach to escalate and de-escalate therapies, combined with continuous control of their efficacy, is still debated. This strategy should follow the individual pathophysiology of traumatic brain injury according to the brain-blood barrier injury. While the management of treatment escalation in TBI by several consensus conferences and guidelines is almost warranted, the tapering of therapies is still under debate and remains challenging. Further studies are necessary to define the best de-escalation management and to refine the current staircase approach; novel pathophysiological considerations may yet provide the ultimate answer.

## Author Contributions

DB and PA wrote the manuscript. DB, PA, PR, IB, AP, GZ, PP, and PF made substantial contributions to the revision, conception, and design of the manuscript. All authors read and approved the final version of the manuscript.

## Conflict of Interest

The authors declare that the research was conducted in the absence of any commercial or financial relationships that could be construed as a potential conflict of interest. The Handling Editor declared a shared affiliation, with no collaboration, with the authors GZ, PF. The Handling Editor declared a past co-authorship with one of the authors DB.

## Abbreviations

AQP4: aquaporin-4
BBB: blood-brain barrier
BTF: Brain Trauma Foundation
CBF: cerebral blood flow
CMRO_2_: cerebral metabolic rate of oxygen
CPP: cerebral perfusion pressure
CSF: cerebral spinal fluid
CT: computed tomography
DC: decompressive craniectomy
EEG: electroencephalographic
ETCO_2_: end-tidal carbon dioxide
EVD: external ventricular drainage
FiO_2_: fraction of inspired oxygen
GABA: gamma-aminobutyric acid
GCS: Glasgow coma scale
HTS: hypertonic saline
HICP: intracranial hypertension
ICP: intracranial pressure
Inv-ICP: invasive intracranial pressure
MAP: mean arterial pressure
MRI: magnetic resonance imaging
NWT: neurologic wake-up test
PaCO_2_: partial pressure of carbon dioxide
PaO_2_: partial pressure of oxygen
PbtO_2_: brain tissue oxygen tension
SpO_2_: peripheral saturation of oxygen
TBI: traumatic brain injury
THAM: tromethamine
VPS: ventriculoperitoneal shunt.

## References

[1] Hutchinson PJ Kolias AG Tajsic T Adeleye A Aklilu AT Apriawan T. Consensus statement from the international consensus meeting on the role of decompressive craniectomy in the management of traumatic brain injury: consensus statement. Acta Neurochir. (2019) 161:1261–74. 10.1007/s00701-019-03936-y 31134383 PMC6581926

[2] Hutchinson PJ Kolias AG Timofeev IS Corteen EA Czosnyka M Timothy J. Trial of decompressive craniectomy for traumatic intracranial hypertension. N Engl J Med. (2016) 375:1119–30. 10.1056/NEJMoa1605215 27602507

[3] Stocchetti N Maas AIR. Traumatic intracranial hypertension. N Engl J Med. (2014) 370:2121–30. 10.1056/NEJMra1208708 24869722

[4] Carney N Totten AM O'Reilly C Ullman JS Hawryluk GWJ Bell MJ. Guidelines for the management of severe traumatic brain injury, Fourth Edition. Neurosurgery. (2017) 80:6–15. 10.1227/NEU.0000000000001432 27654000

[5] Chesnut RM Temkin N Carney N Dikmen S Rondina C Videtta W. A trial of intracranial-pressure monitoring in traumatic brain injury. N Engl J Med. (2012) 367:2471–81. 10.1056/NEJMoa1207363 23234472 PMC3565432

[6] Chesnut RM Temkin N Videtta W Petroni G Lujan S Pridgeon J Consensus-based management protocol (CREVICE Protocol) for the treatment of severe traumatic brain injury based on imaging and clinical examination for use when intracranial pressure monitoring is not employed. J Neurotrauma. (2020) 37:1291–9. 10.1089/neu.2017.5599 32013721 PMC7249475

[7] Aiolfi A Khor D Cho J Benjamin E Inaba K Demetriades D. Intracranial pressure monitoring in severe blunt head trauma: does the type of monitoring device matter? J Neurosurg. (2018) 128:828–33. 10.3171/2016.11.JNS162198 28548592

[8] Chesnut R Aguilera S Buki A Bulger E Citerio G Cooper DJ. A management algorithm for adult patients with both brain oxygen and intracranial pressure monitoring: the seattle international severe traumatic brain injury consensus conference (SIBICC). Intensive Care Med. (2020) 46:919–29. 10.1007/s00134-019-05900-x 31965267 PMC7210240

[9] Hawryluk GWJ Aguilera S Buki A Bulger E Citerio G Cooper DJ. A management algorithm for patients with intracranial pressure monitoring: the Seattle International severe traumatic brain injury consensus conference (SIBICC). Intensive Care Med. (2019) 45:1783–94. 10.1007/s00134-019-05805-9 31659383 PMC6863785

[10] Chesnut R Videtta W Vespa P Le Roux P. Participants in the International Multidisciplinary Consensus Conference on Multimodality Monitoring. Intracranial pressure monitoring: fundamental considerations and rationale for monitoring. Neurocrit Care. (2014) 21(Suppl 2):S64–84. 10.1007/s12028-014-0048-y 25208680

[11] Alali AS Temkin N Barber J Pridgeon J Chaddock K Dikmen S. A clinical decision rule to predict intracranial hypertension in severe traumatic brain injury. J Neurosurg. (2019) 131:612–9. 10.3171/2018.4.JNS173166 30265194 PMC6586526

[12] Eisenberg HM Frankowski RF Contant CF Marshall LF Walker MD. High-dose barbiturate control of elevated intracranial pressure in patients with severe head injury. J Neurosurg. (1988) 69:15–23. 10.3171/jns.1988.69.1.0015 3288723

[13] Cooper DJ Rosenfeld JV Murray L Arabi YM Davies AR D'Urso P. Decompressive craniectomy in diffuse traumatic brain injury. N Engl J Med. (2011) 364:1493–502. 10.1056/NEJMoa1102077 21434843

[14] Censullo JL Sebastian S. Pentobarbital sodium coma for refractory intracranial hypertension. J Neurosci Nurs. (2003) 35:252–62. 10.1097/01376517-200310000-00003 14593936

[15] Robba C Citerio G. How I manage intracranial hypertension. Crit Care. (2019) 23:243. 10.1186/s13054-019-2529-z 31272474 PMC6611036

[16] Bugedo G Santis C. Intracranial hypertension and deep sedation. Crit Care. (2019) 23:342. 10.1186/s13054-019-2578-3 31684988 PMC6827174

[17] Oddo M Crippa IA Mehta S Menon D Payen J-F Taccone FS Optimizing sedation in patients with acute brain injury. Crit Care. (2016) 20:128 10.1186/s13054-016-1294-5 27145814 PMC4857238

[18] Laffey JG Kavanagh BP. Hypocapnia. N Engl J Med. (2002) 347:43–53. 10.1056/NEJMra012457 12097540

[19] Albanèse J Garnier F Bourgoin A Léone M. The agents used for sedation in neurointensive care unit. Ann Fr Anesth Reanim. (2004) 23:528–34. 10.1016/j.annfar.2004.01.010 15158248

[20] Wang X Ding X Tong Y Zong J Zhao X Ren H Ketamine does not increase intracranial pressure compared with opioids: meta-analysis of randomized controlled trials. J Anesth. (2014) 28:821–7. 10.1007/s00540-014-1845-3 24859931

[21] Roberts DJ Hall RI Kramer AH Robertson HL Gallagher CN Zygun DA. Sedation for critically ill adults with severe traumatic brain injury: a systematic review of randomized controlled trials^*^. Crit Care Med. (2011) 39:2743–51. 10.1097/CCM.0b013e318228236f 22094498

[22] Hutchens MP Memtsoudis S Sadovnikoff N. Propofol for sedation in neuro-intensive care. Neurocrit Care. (2006) 4: 54–62. 10.1385/NCC:4:1:054 16498196

[23] Bauer TM Ritz R Haberthür C Haefeli WE Scollo-Lavizzari G Ha HR. Prolonged sedation due to accumulation of conjugated metabolites of midazolam. Lancet. (1995) 346:145–7. 10.1016/S0140-6736(95)91209-6 7603229

[24] Himmelseher S Durieux ME. Revising a dogma: ketamine for patients with neurological injury? Anesth Analg. (2005) 101:524–34. 10.1213/01.ANE.0000160585.43587.5B 16037171

[25] Ropper AH. Hyperosmolar therapy for raised intracranial pressure. N Engl J Med. (2012) 367:746–52. 10.1056/NEJMct1206321 22913684

[26] Marmarou A. The pathophysiology of brain edema and elevated intracranial pressure. Cleve Clin J Med. (2004) 71:S6. 10.3949/ccjm.71.Suppl_1.S6 14964471

[27] Kwiecien JM Dabrowski W Dabrowska-Bouta B Sulkowski G Oakden W Kwiecien-Delaney CJ. Prolonged inflammation leads to ongoing damage after spinal cord injury. PLoS ONE. (2020) 15:e0226584. 10.1371/journal.pone.0226584 32191733 PMC7081990

[28] Saadoun S Papadopoulos MC. Aquaporin-4 in brain and spinal cord oedema. Neuroscience. (2010) 168:1036–46. 10.1016/j.neuroscience.2009.08.019 19682555

[29] Nesic O Lee J Ye Z Unabia GC Rafati D Hulsebosch CE. Acute and chronic changes in aquaporin 4 expression after spinal cord injury. Neuroscience. (2006) 143:779–92. 10.1016/j.neuroscience.2006.08.079 17074445 PMC1894918

[30] Xu Y Yao H Wang Q Xu W Liu K Zhang J. Aquaporin-3 attenuates oxidative stress-induced nucleus pulposus cell apoptosis through regulating the P38 MAPK pathway. Cell Physiol Biochem. (2018) 50:1687–97. 10.1159/000494788 30384362

[31] Nomani AZ Nabi Z Rashid H Janjua J Nomani H Majeed A. Osmotic nephrosis with mannitol: review article. Ren Fail. (2014) 36:1169–76. 10.3109/0886022X.2014.926758 24941319

[32] Hays AN Lazaridis C Neyens R Nicholas J Gay S Chalela JA. Osmotherapy: use among neurointensivists. Neurocrit Care. (2011) 14:222–8. 10.1007/s12028-010-9477-4 21153930

[33] Jagannatha AT Sriganesh T Devi BI Rao GSU. An equiosmolar study on early intracranial physiology and long term outcome in severe traumatic brain injury comparing mannitol and hypertonic saline. J Clin Neurosci. (2016) 27:68–73. 10.1016/j.jocn.2015.08.035 26924183

[34] Mangat HS Chiu Y-L Gerber LM Alimi M Ghajar J Härtl R. Hypertonic saline reduces cumulative and daily intracranial pressure burdens after severe traumatic brain injury. J Neurosurg. (2015) 122:202–210. 10.3171/2014.10.JNS132545 25380107

[35] Major EH O'Connor P Mullan B. Single bolus 30 % hypertonic saline for refractory intracranial hypertension. Irish J Med Sci. (2015) 184:159–65. 10.1007/s11845-014-1080-9 24532091

[36] Colton K Yang S Hu PF Chen HH Stansbury LG Scalea TM. Responsiveness to therapy for increased intracranial pressure in traumatic brain injury is associated with neurological outcome. Injury. (2014) 45:2084–8. 10.1016/j.injury.2014.08.041 25304159

[37] Dias C Silva MJ Pereira E Silva S Cerejo A Smielewski P. Post-traumatic multimodal brain monitoring: response to hypertonic saline. J Neurotrauma. (2014) 31:1872–80. 10.1089/neu.2014.3376 24915462

[38] Ichai C Payen J-F Orban J-C Quintard H Roth H Legrand R. Half-molar sodium lactate infusion to prevent intracranial hypertensive episodes in severe traumatic brain injured patients: a randomized controlled trial. Intensive Care Med. (2013) 39:1413–22. 10.1007/s00134-013-2978-9 23749153

[39] Roquilly A Mahe P Latte D Loutrel O Champin P Di Falco C. Continuous controlled-infusion of hypertonic saline solution in traumatic brain-injured patients: a 9-year retrospective study. Crit Care. (2011) 15:R260. 10.1186/cc10522 22035596 PMC3334811

[40] Eskandari R Filtz MR Davis GE Hoesch RE. Effective treatment of refractory intracranial hypertension after traumatic brain injury with repeated boluses of 14.6% hypertonic saline. J Neurosurg. (2013) 119:338–46. 10.3171/2013.4.JNS121541 23706055

[41] Diringer MN Scalfani MT Zazulia AR Videen TO Dhar R Powers WJ. Effect of mannitol on cerebral blood volume in patients with head injury. Neurosurgery. (2012) 70:1215–9. 10.1227/NEU.0b013e3182417bc2 22089753 PMC3727970

[42] Wells DL Swanson JM Wood GC Magnotti LJ Boucher BA Croce MA. The relationship between serum sodium and intracranial pressure when using hypertonic saline to target mild hypernatremia in patients with head trauma. Crit Care. (2012) 16:R193. 10.1186/cc11678 23068293 PMC3682295

[43] Scalfani MT Dhar R Zazulia AR Videen TO Diringer MN. Effect of osmotic agents on regional cerebral blood flow in traumatic brain injury. J Crit Care. (2012) 27:526.e7-526.e12. 10.1016/j.jcrc.2011.10.008 22176808 PMC3310941

[44] Paredes-Andrade E Solid CA Rockswold SB Odland RM Rockswold GL. Hypertonic saline reduces intracranial hypertension in the presence of high serum and cerebrospinal fluid osmolalities. Neurocrit Care. (2012) 17:204–10. 10.1007/s12028-011-9574-z 21725694

[45] Sakellaridis N Pavlou E Karatzas S Chroni D Vlachos K Chatzopoulos K. Comparison of mannitol and hypertonic saline in the treatment of severe brain injuries. J Neurosurg. (2011) 114:545–8. 10.3171/2010.5.JNS091685 21087203

[46] Bourdeaux CP Brown JM. Randomized controlled trial comparing the effect of 8.4% sodium bicarbonate and 5% sodium chloride on raised intracranial pressure after traumatic brain injury. Neurocrit Care. (2011) 15:42–5. 10.1007/s12028-011-9512-0 21298358

[47] Rhind SG Crnko NT Baker AJ Morrison LJ Shek PN Scarpelini S. Prehospital resuscitation with hypertonic saline-dextran modulates inflammatory, coagulation and endothelial activation marker profiles in severe traumatic brain injured patients. J Neuroinflammation. (2010) 7:5. 10.1186/1742-2094-7-5 20082712 PMC2819256

[48] Oddo M Levine JM Frangos S Carrera E Maloney-Wilensky E Pascual JL. Effect of mannitol and hypertonic saline on cerebral oxygenation in patients with severe traumatic brain injury and refractory intracranial hypertension. J Neurol Neurosurg Psychiatry. (2009) 80:916–20. 10.1136/jnnp.2008.156596 19293171

[49] Kerwin AJ Schinco MA Tepas JJ Renfro WH Vitarbo EA Muehlberger M. The use of 23.4% hypertonic saline for the management of elevated intracranial pressure in patients with severe traumatic brain injury: a pilot study. J Trauma Inj Infect Crit Care. (2009) 67:277–82. 10.1097/TA.0b013e3181acc726 19667879

[50] Ichai C Armando G Orban J-C Berthier F Rami L Samat-Long C. Sodium lactate versus mannitol in the treatment of intracranial hypertensive episodes in severe traumatic brain-injured patients. Intensive Care Med. (2009) 35:471–9. 10.1007/s00134-008-1283-5 18807008

[51] Froelich M Ni Q Wess C Ougorets I Härtl R. Continuous hypertonic saline therapy and the occurrence of complications in neurocritically ill patients^*^. Crit Care Med. (2009) 37:1433–41. 10.1097/CCM.0b013e31819c1933 19242317

[52] Rockswold GL Solid CA Paredes-Andrade E Rockswold SB Jancik JT Quickel RR. Hypertonic saline and its effect on intracranial pressure, cerebral perfusion pressure, and brain tissue oxygen. Neurosurgery. (2009) 65:1035–42. 10.1227/01.NEU.0000359533.16214.04 19934962

[53] Francony G Fauvage B Falcon D Canet C Dilou H Lavagne P. Equimolar doses of mannitol and hypertonic saline in the treatment of increased intracranial pressure^*^. Crit Care Med. (2008) 36:795–800. 10.1097/CCM.0B013E3181643B41 18209674

[54] Sorani MD Morabito D Rosenthal G Giacomini KM Manley GT. Characterizing the dose-response relationship between mannitol and intracranial pressure in traumatic brain injury patients using a high-frequency physiological data collection system. J Neurotrauma. (2008) 25:291–8. 10.1089/neu.2007.0411 18373479

[55] Sakowitz OW Stover JF Sarrafzadeh AS Unterberg AW Kiening KL. Effects of mannitol bolus administration on intracranial pressure, cerebral extracellular metabolites, and tissue oxygenation in severely head-injured patients. J Trauma Inj Infect Crit Care. (2007) 62:292–8. 10.1097/01.ta.0000203560.03937.2d 17297315

[56] Soustiel JF Mahamid E Chistyakov A Shik V Benenson R Zaaroor M. Comparison of moderate hyperventilation and mannitol for control of intracranial pressure control in patients with severe traumatic brain injury – a study of cerebral blood flow and metabolism. Acta Neurochir. (2006) 148:845–51. 10.1007/s00701-006-0792-7 16763735

[57] Ware ML Nemani VM Meeker M Lee C Morabito DJ Manley GT. Effects of 23.4% sodium chloride solution in reducing intracranial pressure in patients with traumatic brain injury: a preliminary study. Neurosurgery. (2005) 57:727–36; discussion 727–36. 16239885

[58] Gasco J Sendra J Lim J Ng I. Linear correlation between stable intracranial pressure decrease and regional cerebral oxygenation improvement following mannitol administration in severe acute head injury patients. In: Intracranial Pressure and Brain Monitoring XII. Acta Neurochirurgica Supplementum, Vol 95. Springer, Vienna (2005). Available online at: 10.1007/3-211-32318-X_16 16463824

[59] Munar F Ferrer AM De Nadal M Poca MA Pedraza S Sahuquillo J. Cerebral hemodynamic effects of 7.2% hypertonic saline in patients with head injury and raised intracranial pressure. J Neurotrauma. (2000) 17:41–51. 10.1089/neu.2000.17.41 10674757

[60] Horn P Münch E Vajkoczy P Herrmann P Quintet M Schilling L. Hypertonic saline solution for control of elevated intracranial pressure in patients with exhausted response to mannitol and barbiturates. Neurol Res. (1999) 21:758–64. 10.1080/01616412.1999.11741010 10596385

[61] Suarez JI Qureshi AI Bhardwaj A Williams MA Schnitzer MS Mirski M. Treatment of refractory intracranial hypertension with 23.4% saline. Crit Care Med. (1998) 26:1118–22. 10.1097/00003246-199806000-00038 9635664

[62] Haärtl R Bardt TF Kiening KL Sarrafzadeh AS Schneider G-H Unterberg AW. Mannitol decreases ICP but does not improve brain-tissue pO2 in severely head-injured patients with intracranial hypertension. Acta Neurochir Suppl. (1997) 70:40–2. 10.1007/978-3-7091-6837-0_12 9416272

[63] Härtl R Ghajar J Hochleuthner H Mauritz W. Hypertonk/hyperoncotic saline reliably reduces ICP in severely head-injured patients with intracranial hypertension. Acta Neurochir Suppl. (1997) 70:126–9. 10.1007/978-3-7091-6837-0_39 9416299

[64] Unterberg AW Kiening KL Hartl R Bardt T Sarrafzadeh AS Lanksch WR. Multimodal monitoring in patients with head injury. J Trauma Inj Infect Crit Care. (1997) 42:32S–37S. 10.1097/00005373-199705001-00006 9191693

[65] Fortune JB Feustel PJ Graca L Hasselbarth J Kuehler DH. Effect of hyperventilation, mannitol, and ventriculostomy drainage on cerebral blood flow after head injury. J Trauma Inj Infect Crit Care. (1995) 39:1091–9. 10.1097/00005373-199512000-00014 7500400

[66] Kimura A Hsu M Seldin M Verkman AS Scharfman HE Binder DK. Protective role of aquaporin-4 water channels after contusion spinal cord injury. Ann Neurol. (2010) 67:794–801. 10.1002/ana.22023 20517941

[67] Sterns RH. Disorders of plasma sodium — causes, consequences, and correction. N Engl J Med. (2014) 372:55–65. 10.1056/NEJMra1404489 25551526

[68] Chen H Song Z Dennis JA. Hypertonic saline versus other intracranial pressure-lowering agents for people with acute traumatic brain injury. Cochrane Database Syst Rev. (2020) 1:CD010904. 10.1002/14651858.CD010904.pub3 31978260 PMC6984412

[69] Stopa BM Dolmans RGF Broekman MLD Gormley WB Mannix R Izzy S. Hyperosmolar therapy in pediatric severe traumatic brain injury—a systematic review. Crit Care Med. (2019) 47:e1022–031. 10.1097/CCM.0000000000004003 31567404

[70] DeNett T Feltner C Hypertonic saline versus mannitol for the treatment of increased intracranial pressure in traumatic brain injury. J Am Assoc Nurse Pract. (2019) 30:S12–8. 10.1097/JXX.0000000000000340 31809399

[71] Bales JW Bonow RH Buckley RT Barber J Temkin N Chesnut RM. Primary external ventricular drainage catheter versus intraparenchymal ICP monitoring: outcome analysis. Neurocrit Care. (2019) 31:11–21. 10.1007/s12028-019-00712-9 31037639

[72] Lundberg N. Continuous recording and control of ventricular fluid pressure in neurosurgical practice. Acta Psychiatr Scand Suppl. (1960) 36:1–193. 13764297

[73] Guillaume J Janny P. Continuous intracranial manometry; importance of the method and first results. Rev Neurol. (1951) 84:131–42. 14845379

[74] Bratton SL Chestnut RM Ghajar J McConnell Hammond FF Harris OA Hartl R. VII. Intracranial pressure monitoring technology. J Neurotrauma. (2007) 24:S-45–S-54. 10.1089/neu.2007.9989 17511545

[75] Nag DS Sahu S Swain A Kant S. Intracranial pressure monitoring: Gold standard and recent innovations. World J Clin Cases. (2019) 7:1535–53. 10.12998/wjcc.v7.i13.1535 31367614 PMC6658373

[76] Park Y-G Woo H-J Kim E Park J. Accuracy and safety of bedside external ventricular drain placement at two different cranial sites: Kocher's point versus forehead. J Korean Neurosurg Soc. (2011) 50:317. 10.3340/jkns.2011.50.4.317 22200013 PMC3243834

[77] Friedman WA Vries JK. Percutaneous tunnel ventriculostomy. J Neurosurg. (1980) 53:662–5. 10.3171/jns.1980.53.5.0662 7431075

[78] Saladino A White JB Wijdicks EFM Lanzino G. Malplacement of ventricular catheters by neurosurgeons: a single institution experience. Neurocrit Care. (2009) 10:248–52. 10.1007/s12028-008-9154-z 18923816

[79] Bhatia A Gupta AK Neuromonitoring in the intensive care unit. I. Intracranial pressure and cerebral blood flow monitoring. Intensive Care Med. (2007) 33:1263–71. 10.1007/s00134-007-0678-z 17522844

[80] Bauer DF Razdan SN Bartolucci AA Markert JM. Meta-analysis of hemorrhagic complications from ventriculostomy placement by neurosurgeons. Neurosurgery. (2011) 69:255–60. 10.1227/NEU.0b013e31821a45ba 21471831

[81] Gelabert-González M Ginesta-Galan V Sernamito-García R Allut AG Bandin-Diéguez J Rumbo RM. The camino intracranial pressure device in clinical practice. assessment in a 1000 cases. Acta Neurochir. (2006) 148:435–41. 10.1007/s00701-005-0683-3 16374566

[82] Al-Tamimi YZ Helmy A Bavetta S Price SJ. Assessment of zero drift in the Codman intracranial pressure monitor: a study from 2 neurointensive care units. Neurosurgery. (2009) 64:94–8; discussion 98-9. 10.1227/01.NEU.0000328392.98602.5A 19145157

[83] Schuürer L Muünch E Piepgras A Weigel R Schilling L Schmiedek P 1700 assessment of the CAMINO intracranial pressure device in clinical 1701 practice. Acta Neurochir Suppl. (1997) 70:296–8. 10.1007/978-3-7091-6837-0_92 9416352

[84] Binz DD Toussaint LG Friedman JA. Hemorrhagic complications of ventriculostomy placement: a meta-analysis. Neurocrit Care. (2009) 10:253. 10.1007/s12028-009-9193-0 19224404

[85] Chatzi M Karvouniaris M Makris D Tsimitrea E Gatos C Tasiou A. Bundle of measures for external cerebral ventricular drainage-associated ventriculitis^*^. Crit Care Med. (2014) 42:66–73. 10.1097/CCM.0b013e31829a70a5 23982025

[86] Volovici V Huijben JA Ercole A Stocchetti N Dirven CMF van der Jagt M. Ventricular drainage catheters versus intracranial parenchymal catheters for intracranial pressure monitoring-based management of traumatic brain injury: a systematic review and meta-analysis. J Neurotrauma. (2019) 36:988–95. 10.1089/neu.2018.6086 30251919

[87] Liu H Wang W Cheng F Yuan Q Yang J Hu J. External ventricular drains versus intraparenchymal intracranial pressure monitors in traumatic brain injury: a prospective observational study. World Neurosurg. (2015) 83:794–800. 10.1016/j.wneu.2014.12.040 25541084

[88] Kasotakis G Michailidou M Bramos A Chang Y Velmahos G Alam H. Intraparenchymal vs extracranial ventricular drain intracranial pressure monitors in traumatic brain injury: less is more? J Am Coll Surg. (2012) 214:950–7. 10.1016/j.jamcollsurg.2012.03.004 22541986

[89] Robba C Cardim D Tajsic T Pietersen J Bulman M Donnelly J. Ultrasound non-invasive measurement of intracranial pressure in neurointensive care: a prospective observational study. PLOS Med. (2017) 14:e1002356. 10.1371/journal.pmed.1002356 28742869 PMC5526499

[90] Godoy DA Seifi A Garza D Lubillo-Montenegro S Murillo-Cabezas F. Hyperventilation therapy for control of posttraumatic intracranial hypertension. Front Neurol. (2017) 8:250. 10.3389/fneur.2017.00250 28769857 PMC5511895

[91] Diringer MN Yundt K Videen TO Adams RE Zazulia AR Deibert E. No reduction in cerebral metabolism as a result of early moderate hyperventilation following severe traumatic brain injury. J Neurosurg. (2000) 92:7–13. 10.3171/jns.2000.92.1.0007 10616076

[92] Roberts BW Karagiannis P Coletta M Kilgannon JH Chansky ME Trzeciak S. Effects of PaCO2 derangements on clinical outcomes after cerebral injury: a systematic review. Resuscitation. (2015) 91:32–41. 10.1016/j.resuscitation.2015.03.015 25828950

[93] Zhang Z Guo Q Wang E. Hyperventilation in neurological patients. Curr Opin Anaesthesiol. (2019) 32:568–573. 10.1097/ACO.0000000000000764 31211719 PMC6735527

[94] Roberts I Sydenham E. Barbiturates for acute traumatic brain injury. Cochrane Database Syst Rev 2. (2012) 12:CD00003: 10.1002/14651858.CD000033.pub2 23235573 PMC7061245

[95] Muizelaar JP Marmarou A Ward JD Kontos HA Choi SC Becker DP. Adverse effects of prolonged hyperventilation in patients with severe head injury: a randomized clinical trial. J Neurosurg. (1991) 75:731–9. 10.3171/jns.1991.75.5.0731 1919695

[96] Brandi G Stocchetti N Pagnamenta A Stretti F Steiger P Klinzing S. Cerebral metabolism is not affected by moderate hyperventilation in patients with traumatic brain injury. Crit Care. (2019) 23:45. 10.1186/s13054-018-2304-6 30760295 PMC6375161

[97] Roberts I Schierhout G. Hyperventilation therapy for acute traumatic brain injury. Cochrane Database Syst Rev. (1997) 1997:CD000566. 10.1002/14651858.CD000566 10796728 PMC7061354

[98] Cottenceau V Petit L Masson F Guehl D Asselineau J Cochard J-F. The use of bispectral index to monitor barbiturate coma in severely brain-injured patients with refractory intracranial hypertension. Anesth Analg. (2008) 107:1676–82. 10.1213/ane.0b013e318184e9ab 18931232

[99] Stocchetti N Zanaboni C Colombo A Citerio G Beretta L Ghisoni L. Refractory intracranial hypertension and “second-tier” therapies in traumatic brain injury. Intensive Care Med. (2008) 34:461–7. 10.1007/s00134-007-0948-9 18066523

[100] Robles LA Cuevas-Solórzano A. Massive brain swelling and death after cranioplasty: a systematic review. World Neurosurg. (2018) 111:99–108. 10.1016/j.wneu.2017.12.061 29269069

[101] Schalén W Messeter K Nordström C-H. Complications and side effects during thiopentone therapy in patients with severe head injuries. Acta Anaesthesiol Scand. (1992) 36:369–77. 10.1111/j.1399-6576.1992.tb03483.x 1595344

[102] Pérez-Bárcena J Llompart-Pou JA Homar J Abadal JM Raurich JM Frontera G. Pentobarbital versus thiopental in the treatment of refractory intracranial hypertension in patients with traumatic brain injury: a randomized controlled trial. Crit Care. (2008) 12:R112. 10.1186/cc6999 18759980 PMC2575601

[103] Tsoucalas G Kousoulis AA Mariolis-Sapsakos T Sgantzos M. Trepanation practices in asclepieia: systematizing a neurosurgical innovation. World Neurosurg. (2017) 103:501–3. 10.1016/j.wneu.2017.04.022 28419880

[104] Hawryluk GWJ Rubiano AM Totten AM O'Reilly C Ullman JS Bratton SL. Guidelines for the management of severe traumatic brain injury: 2020 update of the decompressive craniectomy recommendations. Neurosurgery. (2020) 87:427–34. 10.1093/neuros/nyaa278 32761068 PMC7426189

[105] Stocchetti N Longhi L Zanier ER. Intracranial pressure monitoring for traumatic brain injury: available evidence and clinical implications. Minerva Anestesiol. (2008) 74:197–203. 18414362

[106] Qiu W Guo C Shen H Chen K Wen L Huang H. Effects of unilateral decompressive craniectomy on patients with unilateral acute post-traumatic brain swelling after severe traumatic brain injury. Crit Care. (2009) 13:R185. 10.1186/cc8178 19930556 PMC2811943

[107] Jiang J-Y Xu W Li W-P Xu W-H Zhang J Bao Y-H. Efficacy of standard trauma craniectomy for refractory intracranial hypertension with severe traumatic brain injury: a multicenter, prospective, randomized controlled study. J Neurotrauma. (2005) 22:623–8. 10.1089/neu.2005.22.623 15941372

[108] Phan K Moore JM Griessenauer C Dmytriw AA Scherman DB Sheik-Ali S. Craniotomy versus decompressive craniectomy for acute subdural hematoma: systematic review and meta-analysis. World Neurosurg. (2017) 101:677–85.e2. 10.1016/j.wneu.2017.03.024 28315797

[109] Zhang D Xue Q Chen J Dong Y Hou L Jiang Y. Decompressive craniectomy in the management of intracranial hypertension after traumatic brain injury: a systematic review and meta-analysis. Sci Rep. (2017) 7:8800. 10.1038/s41598-017-08959-y 28821777 PMC5562822

[110] Zhang K Jiang W Ma T Wu H. Comparison of early and late decompressive craniectomy on the long-term outcome in patients with moderate and severe traumatic brain injury: a meta-analysis. Br J Neurosurg. (2016) 30:251–7. 10.3109/02688697.2016.1139052 26828333

[111] Grindlinger GA Skavdahl DH Ecker RD Sanborn MR. Decompressive craniectomy for severe traumatic brain injury: clinical study, literature review and meta-analysis. Springerplus. (2016) 5:1605. 10.1186/s40064-016-3251-9 27652178 PMC5028365

[112] Tsaousi GG Marocchi L Sergi PG Pourzitaki C Santoro A Bilotta F. Early and late clinical outcomes after decompressive craniectomy for traumatic refractory intracranial hypertension: a systematic review and meta-analysis of current evidence. J Neurosurg Sci. (2020) 64:97–106. 10.23736/S0390-5616.18.04527-7 30356035

[113] Lu G Zhu L Wang X Zhang H Li Y. Decompressive craniectomy for patients with traumatic brain injury: a pooled analysis of randomized controlled trials. World Neurosurg. (2020) 133:e135–48. 10.1016/j.wneu.2019.08.184 31491576

[114] Garg K Singh P Singla R Aggarwal A Borle A Singh M. Role of decompressive craniectomy in traumatic brain injury – a meta-analysis of randomized controlled trials. Neurol India. (2019) 67:1225. 10.4103/0028-3886.271260 31744947

[115] Sahuquillo J Dennis JA. Decompressive craniectomy for the treatment of high intracranial pressure in closed traumatic brain injury. Cochrane Database Syst Rev. (2019) 12:CD003983. 10.1002/14651858.CD003983.pub3 31887790 PMC6953357

[116] Iaccarino C Kolias AG Roumy LG Fountas K Adeleye AO. Cranioplasty following decompressive craniectomy. Front Neurol. (2020) 10:1357. 10.3389/fneur.2019.01357 32063880 PMC7000464

[117] Manfiotto M Mottolese C Szathmari A Beuriat P-A Klein O Vinchon M. Decompressive craniectomy and CSF disorders in children. Child's Nerv Syst. (2017) 33:1751–7. 10.1007/s00381-017-3542-7 29149390

[118] Walcott BP Kwon C-S Sheth SA Fehnel CR Koffie RM Asaad WF. Predictors of cranioplasty complications in stroke and trauma patients. J Neurosurg. (2013) 118:757–62. 10.3171/2013.1.JNS121626 23394335

[119] Mukherjee S Thakur B Haq I Hettige S Martin AJ. Complications of titanium cranioplasty—a retrospective analysis of 174 patients. Acta Neurochir. (2014) 156:989–98. 10.1007/s00701-014-2024-x 24615066

[120] Kandasamy J Yousaf J Mallucci C. Third ventriculostomy in normal pressure hydrocephalus. World Neurosurg. (2013) 79:S22.e1–7. 10.1016/j.wneu.2012.02.008 22381824

[121] Goedemans T Verbaan D van der Veer O Bot M Post R Hoogmoed J. Complications in cranioplasty after decompressive craniectomy: timing of the intervention. J Neurol. (2020) 267:1312–20. 10.1007/s00415-020-09695-6 31953606 PMC7184041

[122] Archavlis E Carvi Y Nievas M. The impact of timing of cranioplasty in patients with large cranial defects after decompressive hemicraniectomy. Acta Neurochir. (2012) 154:1055–62. 10.1007/s00701-012-1333-1 22527574

[123] Malcolm JG Rindler RS Chu JK Chokshi F Grossberg JA Pradilla G. Early cranioplasty is associated with greater neurological improvement: a systematic review and meta-analysis. Neurosurgery. (2018) 82:278–88. 10.1093/neuros/nyx182 28419358

[124] Kiening KL Schoening WN Stover JF Unterberg AW. Continuous monitoring of intracranial compliance after severe head injury: relation to data quality, intracranial pressure and brain tissue PO2. Br J Neurosurg. (2003) 17:311–8. 10.1080/02688690310001601199 14579896

[125] Schomer KJ Sebat CM Adams JY Duby JJ Shahlaie K Louie EL. Dexmedetomidine for refractory intracranial hypertension. J Intensive Care Med. (2019) 34:62–6. 10.1177/0885066616689555 28122469

[126] Marklund N. The Neurological wake-up test—a role in neurocritical care monitoring of traumatic brain injury patients? Front Neurol. (2017) 8:540. 10.3389/fneur.2017.00540 29089921 PMC5650971

[127] Herzer G Mirth C Illievich UM Voelckel WG Trimmel H. Analgosedation of adult patients with elevated intracranial pressure. Wien Klin Wochenschr. (2018) 130:45–53. 10.1007/s00508-017-1228-5 28733841

[128] Poca M-A Sahuquillo J Arribas M Báguena M Amorós S Rubio E. Fiberoptic intraparenchymal brain pressure monitoring with the camino V420 monitor: reflections on our experience in 163 severely head-injured patients. J Neurotrauma. (2002) 19:439–48. 10.1089/08977150252932398 11990350

[129] Rebuck JA Murry KR Rhoney DH Michael DB Coplin WM. Infection related to intracranial pressure monitors in adults: analysis of risk factors and antibiotic prophylaxis. J Neurol Neurosurg Psychiatry. (2000) 69:381–4. 10.1136/jnnp.69.3.381 10945814 PMC1737112

[130] Staykov D Kuramatsu JB Bardutzky J Volbers B Gerner ST Kloska SP. Efficacy and safety of combined intraventricular fibrinolysis with lumbar drainage for prevention of permanent shunt dependency after intracerebral hemorrhage with severe ventricular involvement: a randomized trial and individual patient data meta-analysi. Ann Neurol. (2017) 81:93–103. 10.1002/ana.24834 27888608

[131] Oliveira MF de Reis RC Trindade EM Pinto FCG. Evidences in the treatment of idiopathic normal pressure hydrocephalus. Rev Assoc Med Bras. (2015) 61:258–62. 10.1590/1806-9282.61.03.258 26248249

[132] Winfield JA Rosenthal P Kanter RK Casella G. Duration of intracranial pressure monitoring does not predict daily risk of infectious complications. Neurosurgery. (1993) 33:424–30; discussion 430-1. 10.1227/00006123-199309000-00011 8413873

[133] Allin D Czosnyka M Czosnyka Z. Laboratory testing of the Pressio intracranial pressure monitor. Neurosurgery. (2008) 62:1158–61; discussion 1161. 10.1227/01.neu.0000325878.67752.eb 18580814

[134] Prior A D'Andrea A Robba C Fiaschi P Letter to the editor regarding “first intracranial pressure monitoring or first operation: which one is better?”. World Neurosurg. (2020) 13;S1878-8750(20)30690-2 10.1016/j.wneu.2020.03.219 32298830

[135] Kanter RK Weiner LB Patti AM Robson LK. Infectious complications and duration of intracranial pressure monitoring. Crit Care Med. (1985) 13:837–9. 10.1097/00003246-198510000-00012 4028754

[136] Lescot T Bonnet MP Zouaoui A Muller JC Fetita C Coriat P Puybasset L. A quantitative computed tomography assessment of brain weight, volume, and specific gravity in severe head trauma. Intensive Care Med. (2005) 31:1042–50. 10.1007/s00134-005-2709-y 15991008

[137] Cook AM Morgan Jones G Hawryluk GWJ Mailloux P McLaughlin D Papangelou A. Guidelines for the acute treatment of cerebral edema in neurocritical care patients. Neurocrit Care. (2020) 32:647–66. 10.1007/s12028-020-00959-7 32227294 PMC7272487

[138] Degos V Lescot T Icke C Le Manach Y Fero K Sanchez P. Computed tomography-estimated specific gravity at hospital admission predicts 6-month outcome in mild-to-moderate traumatic brain injury patients admitted to the intensive care unit. Anesth Analg. (2012) 114:1026–33. 10.1213/ANE.0b013e318249fe7a 22366842

[139] Barzó P Marmarou A Fatouros P Corwin F Dunbar J. Magnetic resonance imaging-monitored acute blood-brain barrier changes in experimental traumatic brain injury. J Neurosurg. (1996) 85:113–21. 10.3171/jns.1996.85.6.1113 8929504

[140] Degos V Pereira AR Lescot T Sanchez-Peña P Daoudi M Zouaoui A. Does brain swelling increase estimated specific gravity? Neurocrit Care. (2008) 9:338–43. 10.1007/s12028-008-9131-6 18818888

[141] Barzó P Marmarou A Fatouros P Hayasaki K Corwin F. Contribution of vasogenic and cellular edema to traumatic brain swelling measured by diffusion-weighted imaging. J Neurosurg. (1997) 87:900–7. 10.3171/jns.1997.87.6.0900 9384402

[142] Rosner MJ Rosner SD Johnson AH. Cerebral perfusion pressure: management protocol and clinical results. J Neurosurg. (1995) 83:949–62. 10.3171/jns.1995.83.6.0949 7490638

[143] Lescot T Degos V Zouaoui A Préteux F Coriat P Puybasset L. Opposed effects of hypertonic saline on contusions and noncontused brain tissue in patients with severe traumatic brain injury. Neurol Crit Care. (2006) 34:12. 10.1097/01.CCM.0000243797.42346.64 16971850

[144] Grände PO. Critical evaluation of the lund concept for treatment of severe traumatic head injury, 25 years after its introduction. Front Neurol. (2017) 8:315. 10.3389/fneur.2017.00315 28725211 PMC5495987

